# Supplementary material for: Horizontal acquisition of a patchwork Calvin cycle by symbiotic and free-living Campylobacterota (formerly Epsilonproteobacteria)
Source: ISME J. 2019 Sep 27;14(1):104–22. doi: 10.1038/s41396-019-0508-7 (PMC6908604; doi:10.1038/s41396-019-0508-7)
Supplement: Supplementary file 1 — Supplementary Information [file 41396_2019_508_MOESM1_ESM.docx]

# SI appendix

**Contents**

SI appendix note 1 – Description of “*Candidatus* Thiobarbaceae” and its members

SI appendix note 2 – “*Ca*. Thiobarba” genome descriptions

SI appendix note 3 - Assembly results

SI appendix note 4 – Gene cluster organisation

SI appendix Figures S1-S25

Overview of the supplementary tables

Supplementary references

### SI appendix note 1 - Description of “*Candidatus* Thiobarbaceae” and its members

The *Candidatus* family name “Thiobarbaceae” and genus name “Thiobarba” were chosen to describe morphological and metabolic features of the bacteria characterized in this study. As the bacterial epibionts are sulfur-oxidizing filamentous bacteria that colonize the gill epithelia of bathymodiolin mussels in thick mats, we named them “*Thio-*” from the Greek word θεῖον, theîon for sulfur and “*barba*” from the Latin word for beard (sulfur beard).

The *Candidatus* species name “*Ca*. Thiobarba azoricus” and “*Ca*. Thiobarba childressi” were chosen because of the respective animal hosts, *Bathymodiolus azoricus* and *“Bathymodiolus” childressi*, on which they were found.

### SI appendix note 2 – “*Ca.* Thiobarba” genome descriptions

**Metabolism**

An overview of “*Ca*. Thiobarba” is shown in Figure S2.

**Sulfur oxidation**

Both “*Ca.* Thiobarba” genomes had the *soxABCDXYZ* genes, while “*Ca.* T. childressi” also had a flavocytochrome c sulfide dehydrogenase (*fccAB*) and type I sulfide quinone oxidoreductase (*sqr*). These genes are involved in the oxidation of hydrogen sulfide to elemental sulfur, and the transfer of electrons via cytochrome c and menaquinone molecules to the electron transport chain (1–3)*.*

The *fcc* and *sqr* genes were expressed in “*Ca.* T. childressi”, indicating the symbiont's ability to use hydrogen sulfide as an energy source. Hydrogen sulfide is toxic in high concentrations because it inhibits haeme-copper oxidases used for aerobic respiration (4). The ability to oxidize hydrogen sulfide would give “*Ca.* T. childressi” an additional source of energy, while also mitigating the toxic effects of sulfide. Interestingly, neither *sqr* nor *fcc* genes were found in the “*Ca.* T. azoricus” genome. However, *B. azoricus* hosts sulfur-oxidizing endosymbionts, which have these genes. This raises the intriguing possibility that “*Ca.* T. azoricus” epibionts might thus avoid competition with the sulfur-oxidizing endosymbionts for hydrogen sulfide. However, we cannot exclude that the *sqr* and *fcc* genes genes were not found in “*Ca.* T. azoricus” because of the fragmented state of its genome.

**Additional electron donors**

*“Ca.* T. azoricus” and “*Ca.* T. childressi” both had genes to use potential auxiliary electron donors from energy sources other than sulfur oxidation. The “*Ca.* T. azoricus” genome encoded a NiFe uptake hydrogenase and had the operon of accessory genes (*hyp*) required for the maturation of the enzyme (5)*.* When compared to public database, the uptake hydrogenase was most similar to the one present in the campylobacterotum *Arcobacter* sp. LPB0137 with 98% amino acid identity. Hydrogenases generate highly electronegative reductants that the epibiont could use to reduce its pool of quinones (6)*.* The genome of “*Ca.* T. childressi” did not contain hydrogenase genes (although the fragmented assembly of this genome does not allow a definitive statement). Nevertheless, it is intriguing that “*Ca*. T. azoricus”, which is found at hydrothermal vents, has hydrogenases, while the cold seep epibiont may not, because previous studies have found hydrogenases in hydrothermal vent symbioses but not in cold seep associations (7).

“*Ca.* T. childressi” had a formate dehydrogenase complex that may enable it to use formate as an electron donor. This complex is linked to pathway activated when the cell switch from aerobic to anaerobic states suggesting the potential for the “*Ca*. T. childressi” to be a facultative anaerobe (8). Alternatively, it has recently been suggested that formate dehydrogenase-O serves as an electron transfer element in glucose metabolism to promote oxidative stress tolerance and survival in stress situation (9).

The two symbiont genomes encoded quinone reductases, which allow the direct oxidation of quinone using enzymes such as malate:quinone oxidoreductase (*mqo*), succinate dehydrogenase, complex II and III homologs. The presence of a quinol cytochrome c complex III potentially links the oxidation of quinol to the generation of a proton membrane gradient and the reduction of terminal electron acceptors, such as oxygen.

**Electron acceptors**

Both “*Ca.* T. azoricus” and “*Ca.* T. childressi” can use oxygen as a terminal electron acceptor based on the presence of genes encoding cytochrome c oxidase and the low-affinity oxygenase cbb3. “*Ca.* T. azoricus” may be able to respire nitrate. The genes for assimilatory nitrate reduction *napFGBAH* and the large and small nitrite reductase subunit genes were present in the “*Ca.* T. azoricus” genome.

**Oxygen tolerance**

Multiple genes in the epibiont genomes were predicted to enable resistance to oxygen stress such as genes encoding catalases, peroxidases and alkyl hydroperoxide reductases (Ahp). A recent study showed that Ahp was the major antioxidant enzyme in the gammaproteobacterial endosymbiont of the deep-sea tubeworm *Riftia pachyptila* (10). Other metabolic pathways described above that are typical for anaerobic or facultative anaerobic bacteria, such as the oxidation of formate, a byproduct of fermentation, and dissimilatory nitrate reduction highlight the metabolic versatility of the epibionts to adapt to fluctuating oxygen concentrations.

**Central carbon metabolism**

The genomes of both epibionts encoded for glycogenesis, glycolysis, the non-oxidative branch of the pentose phosphate pathway and the oxidative tricarboxylic acid (TCA) cycle. In the glycolysis pathways of both epibionts, the gene encoding 6-phosphofructokinase, which allows the use of sucrose and fructose, was not found, while the non-oxidative branch of the pentose phosphate pathway lacked a transaldolase. Carbon fixed via the CBB cycle was predicted to enter the oxidative TCA cycle through phosphoenolpyruvate and could follow biosynthetic routes to either fumarate or to 2-oxoglutarate. The presence of a 2-methyl citrate cycle was also predicted in both genomes, providing the ability to incorporate propionate into succinate and pyruvate. Pyruvate could then be fermented to acetyl-CoA and used as a cofactor in the TCA cycle. Additionally, oxaloacetate and propionate via the 2-methyl citrate cycle could recharge the pyruvate and succinate pools.

Our genomic predictions indicated that “*Ca*. Thiobarba” have a mixotrophic metabolism while our transcriptomic analyses revealed that genes involved in inorganic carbon fixation were highly expressed. As suggested in another campylobacterotal symbiont (11), both “*Ca*. Thiobarba” likely rely primarily on autotrophic pathways and might switch to a mixotrophic metabolism and use small organic molecules as energy and/or carbon sources, when inorganic carbon becomes limiting such as when the host mussel is away from fluids rich in reduced compounds or when the bacteria is in a potential free living state between hosts.

Intriguingly, the “*Ca.* Thiobarba” genomes encoded many pathways requiring sugars, including N-linked glycosylation, capsular polysaccharides and lipooligosaccharide synthesis, some of which are not found in related chemolithoautotrophic Campylobacterota, but are present in heterotrophic host-associated Campylobacterota. Many of the pathways requiring sugars are predicted to play a role in surface structures and extracellular polysaccharide capsule formation, which can be key mediators of host attachment, and thus may be essential for its epibiotic lifestyle (12, 13).

**Ammonia incorporation**

Both epibionts have the genomic ability to incorporate ammonia using high-affinity ammonium uptake transporters and glutamine synthetase. The later enzyme assimilates ammonia into glutamine with high affinity at very low ammonia concentrations but requires an energy rich environment (14)*.*

**Transporters**

We predicted the presence of transporters in both epibiont genomes, such as ABC-like transporters, which can import sugar, lipids and amino acids into the cytoplasm. These transporters could provide substrate for various metabolic pathways, for example, sugars for glycolysis or glycogenesis and lipids for fatty acid generation or lipopolysaccharide synthesis (15)*.*

Multiple tripartite ATP-independent periplasmic transporters (TRAP transporters) were present in the genomes of both endosymbionts. These transporters belong to a large family of solute transporters found in bacteria and archaea, but not in eukaryotes, that appear to be specific for the uptake of organic acids (14). This transporter family uses a substrate binding protein in combination with a secondary transporter. They have been described to import four carbon molecules, such as malate, fumarate or succinate, into the cytoplasm of bacteria (16). Additionally, they can induce anaplerotic incorporation of fumarate or citrate into different pathways, including the oxidative TCA cycle.

The genes encoding Na+ translocating NADH:quinone oxidoreductase (Na+-NQR) were present in both epibionts. This membrane protein complex couples the oxidation of NADH to generate the export of Na+ into the bacterial periplasm. This efflux maintains a Na+ motive force, which fuels an array of different membrane symporters (17)*.*

**Amino acid and vitamin biosynthesis**

Both “*Ca.* T. azoricus” and “*Ca.* T. childressi” genomes had genes for the synthesis of most amino acids. “*Ca.* T. childressi” lacked genes for phenylalanine synthesis while “*Ca.* T. azoricus” lacked the genes for synthesizing phenylalanine, alanine, proline and selenocysteine. It is not uncommon for bacteria to lack some amino acid synthesis pathways. Also, these genes could be missing in the assembly because of the fragmented nature of both genome drafts. Multiple amino acid transporters were predicted in both epibiont genomes; they could directly import amino acids from the environment.

Both epibionts had some but not all of the genes involved in the *de novo* synthesis of vitamin B6 (pyridoxal 5'-phosphate – PLP) and vitamin B1 (thiamin), which are both dietary requirements for animals. The key genes for PLP synthesis were present and expressed but some of the genes involved in precursor metabolic reactions of this pathway were not found. These compounds could, however, be generated by other metabolic pathways or may have been missing in the assembly. All the genes for the biosynthesis of thiamin were present in “*Ca.* T. childressi”, whereas “*Ca.* T. azoricus” lacked the genes for thiazole biosynthesis, as well as the precursor pathway for the biosynthesis of thiamin. The genes for synthesizing these two vitamins are widespread in bacteria but not in higher eukaryotic organisms (18,19). Their presence in the epibionts could therefore be beneficial for the host’s nutrition.

**Responses to the environment**

Both campylobacterotal epibionts possessed genes that could enable them to adapt to variations in their environment. Their genomes contained a wide array of genes involved in mineral transport, including detoxification mechanisms for heavy metals such as mercury, cobalt, cadmium and copper. Additionally, genes used by prokaryotes to sense and respond to environmental signals were present, such as multiple copies of predicted two-component signal transduction systems and diguanylate cyclase (GGDEF) domains, which are commonly used as molecular messengers triggering specific genetic reactions to environmental stimuli (20–22)*.*

**Chemotaxis and motility**

The genes for a fully functional flagellum were found in both genomes (35 genes). “*Ca.* T. childressi” contained 12 copies of the flagellin gene and “*Ca.* T. azoricus” had 10 copies. Flagellin has been described in campylobacterotal pathogens, such as *Campylobacter* and *Helicobacter*, to be involved in virulence, by promoting motility or adhesion to host cells (23,24)*.* Multiple copies of flagellin genes are common in some bacteria and often associated with phase variation, in which only one copy of a gene is expressed at any given time. Bacteria such as *Campylobacter* use phase variation to switch between different types of flagellin and thus evade the eukaryotic immune system (25)*.*

Chemotaxis-related genes were also present in multiple copies in both epibiont genomes. In chemotaxis, receptor proteins anchored in the bacterial membrane are used for sensing external chemical stimuli (26)*.* These signals are transferred to internal effectors that modulate bacterial motility toward or away from the environmental stimuli. In *Campylobacter* species, chemotaxis is considered an important mechanism in the bacterial colonization of eukaryotic cells by inducing bacteria to move toward the eukaryotic cell (24,27)*.* The chemotaxis receptors methyl chemotaxis protein and transductor were present in 10 copies in “*Ca.* T. childressi” and 4 in “*Ca.* T. azoricus”. Chemotaxis cytoplasmic effector *cheAYVW* genes modulate the excitation/inhibition of the flagellum motor mechanism and were present in 26 copies in “*Ca.* T. childressi” and 10 in “*Ca.* T. azoricus” (27)*.*

**Adhesion and virulence**

Genes involved in N-linked glycosylation, a common protein modification mechanism found in all domains of life, were present in both epibiont genomes. N-linked glycosylation has been extensively studied in the Campylobacterota, especially in *Campylobacter jejuni*, in which it has been shown to play a role in the evasion of the host immune system and in the adhesion and invasion process of the gut epithelium (29)*.* These genes were also found in two Campylobacterota isolated from hydrothermal vents that may be associated with invertebrates, *Sulfurovum litotrophicum* and *Nitratiruptor* sp. (22)*.*

Lipooligosaccharide (LOS) related genes were present in the genomes of both epibionts. In “*Ca.* T. childressi”, the LOS biosynthesis pathway was predicted to be functional, based on the presence of genes for the synthesis of lipid A and O-antigens. Lipid A and O-antigens play a role in the adhesion of pathogens to eukaryotic cells and immune cell evasion. These endotoxins possess a long hydrophobic chain that allows the bacteria to anchor to the eukaryotic host’s cell membrane (30)*.*

Genes for the synthesis of colanic acid were predicted from the genomes of both epibionts. Colanic acid (CA) is a highly viscous capsular polysaccharide. It is a key component of biofilms secreted by Enterobacteria and protects cells under stress, such as exposure to osmotic variation, and it is not involved in pathogenicity (31,32)*.*

Other genes related to virulence or invasion in Campylobacterota were also present in both genomes. Multiple copies of genes encoding fibronectin/fibrinogen binding proteins were annotated in both epibiont genomes. These proteins bind fibronectin and fibrinogen, which are present in the extracellular matrix of eukaryotic epithelia (33)*.* Genes known to play a role in the initial adhesion step of bacteria to their host's gut epithelia (24,34), such as those encoding invasion antigen (CiaB) and paralyzed flagella protein PflA, were also present in both epibionts. In studies in which these genes were inactivated in gastrointestinal pathogens such as *Helicobacter* or *Campylobacter,* the virulence of these bacteria decreased.

### SI appendix note 3 - Assembly results

In contrast to the *B. childressi* metagenomic assembly, where only one Campylobacterota bin was detected, we found three distinct Campylobacterota bins in the metagenomic assemblies from *B. azoricus*. We compared the average nucleotide and amino acid identity (AAI) of these bins with representative Campylobacterota genomes to identify which Campylobacterota family they belong to (Table S2). The first bin was the “*Ca.* T. azoricus” bin related to the *“B.” childressi* Campylobacterota epibiont, the second was most closely related to *Sulfurovum* sp. (AQWF01000016) and the third to *Sulfurimonas* sp. (KC682116). The “*Ca.* T. azoricus” bin had an average coverage of 20X whereas the *Sulfurovum-*related bin and the *Sulfurimonas*-related bins had 5X average coverage.

The *Sulfurovum-* and *Sulfurimonas*-related bins had predicted metabolic pathways commonly found in deep-sea Campylobacterota. They were both predicted to be sulfur oxidizers based on genes encoding the SOX multi-enzyme complex. Only the *Sulfurovum* related bin had all the genes needed for fixing inorganic carbon using the rTCA cycle, whereas the *Sulfurimonas*-related bin was missing one of the key components for the cycle, the genes coding for all subunits of the 2-oxoglutarate oxidoreductase.

In previous FISH studies (35) we found no indications that other Campylobacterota besides “Ca. T. azoricus” were associated with *B. azoricus* gills. Given that *Sulfurovum* and *Sulfurimonas* species are commonly found at hydrothermal vents as free-living and surface-colonizing bacteria, these two bins may have originated from environmental contaminants, although we cannot exclude that those Campylobacterota also colonize *B. azoricus* and were not detected by FISH.

To verify that potential rTCA related genes were not lost during assembly and binning, we extracted 74 ATP-citrate lyase genes (acl), 265 2-oxoglutarate: acceptor oxidoreductase subunits (*oor*) genes and 158 pyruvate flavodoxin/ferredoxin oxidoreductase subunits (*por*) genes from 362 publicly available Campylobacterota genomes in NCBI (list in Table S9) and mapped these reference reads to our two metagenomic libraries. Despite being the deepest-sequenced library (470 million reads for “*B.” childressi* vs. 15 million reads for *B. azoricus*), none of the reads from the “*B.” childressi* metagenome mapped to any of the *oor* or *acl* reference sequences, while 622 reads mapped to the *por* genes. To ensure that these *por* reads were correctly assigned to “*Ca*. Thiobarba childressi”, we re-mapped them to the “*Ca*. T. childressi” genome. All 622 reads mapped to the “*Ca*. Thiobarba childressi” reference sequence, suggesting that we captured the *por* diversity within the “*B.” childressi* metagenomic dataset. This analysis provides additional support that the *oor* and *acl* genes were truly absent and no other *por* genes were missed during assembly and binning.

From the *B. azoricus* metagenome, 1236, 1502 and 2448 reads mapped to the *por*, *oor* and *acl* reference sequences, respectively. To ensure that the reads were correctly assigned to the *Sulfurovum*- and *Sulfurimonas*-related bins, we de novo assembled these reads using SPAdes (36) and compared the assembled genes with the rTCA genes found in the *B. azoricus* metagenomic assembly using BBmap (BBmap suite v37.9). The de novo assembled contigs only contained rTCA genes that were identical to those in the *Sulfurovum*- and *Sulfurinonas*-related bins. We reconstructed four *acl* genes, two *aclA* and two *aclB* genes, which were identical to those present either in *Sulfurimonas*- or *Sulfurovum*-related bins. The *oor* gene reassembly provided six contigs, three too small to annotate, one including a partial *oorABDG* operon identical to the operon present in the *Sulfurovum*-related bin, and two smaller contigs including the genes *oorABG*. The two latter contigs were related to *Sulfurimonas* genomes and had a similar GC content (36 and 38%) to the *Sulfurimonas*-related bin assembled from the *B. azoricus* metagenome (see Fig. S27), suggesting that these might be the *oor* genes that were not found in the *Sulfurimonas*-related bin. Finally, the *por* gene reassembly resulted in 10 contigs: five were too small to be annotated, one mapped to the *Sulfurimonas*-related *porA*, three mapped to the *porABG* genes and the last to the “*Ca.* T. azoricus” *porB* gene.

### SI appendix note 4 – Gene cluster organisation

The organization of the two CBB cycle gene clusters in “*Ca*. Thiobarba” support their evolutionary origin from Beta- and Gammaproteobacteria (SI appendix Fig. S24). Gene order in the cluster affiliated at the sequence level with Gammaproteobacteria was similar to that of *Bathymodiolus* sulfur-oxidizing symbionts and their closest free-living relative “*Ca.* Thioglobus autotrophica” (CP010552). Gene order in the cluster affiliated at the sequence level with Betaproteobacteria was similar to that of autotrophic Betaproteobacteria such as *Dechloromonas aromatica* (NC_007298), *Thiomonas delicata* (NZ_LT592170) and *Paraburkholderia xenovorans* (NC_007951). The phylogeny and gene organization therefore consistently support at least two independent acquisitions of these CBB cycle genes by “*Ca.* Thiobarba”. In the draft genome of the free-living Campylobacterota from diffuse hydrothermal fluids collected in the Manus Basin (25), only the *rbcL*, *rbcS*, *cbbQ* and *cbbO* genes appear to have been acquired horizontally, based on their close phylogenetic relationship to the corresponding genes in Gammaproteobacteri and “*Ca.* Thiobarba”. Other genes involved in the CBB cycle clustered with sequences from other Campylobacterota, with the exception of those encoding fructose bisphosphatase aldolase and the ribulose phosphate 3-epimerase, which were present in a second copy closely related but not identical to the “*Ca.* Thiobarba” genes (SI appendix Fig. S9 and S12).

### Supplementary information figures


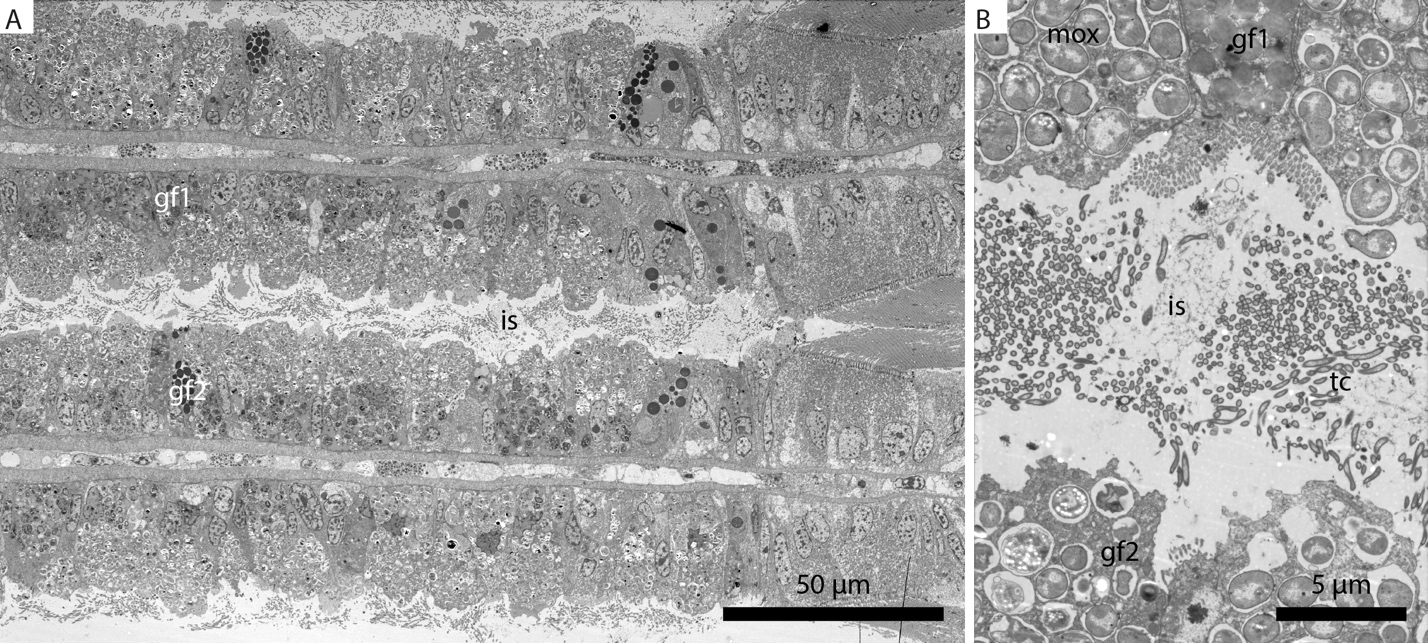


**Figure S1**: Electron micrograph of “*Bathymodiolus” childressi* gill filament cross-section.
(A) shows two gill filaments (gf1 and gf2) with the inter-filament space (is) between them. (B) shows a higher magnification of the two gill filaments that harbor the methane-oxidizing endosymbiont (mox), and the inter-filament space between them where “*Ca*. Thiobarba childressi” (tc) is located.

###
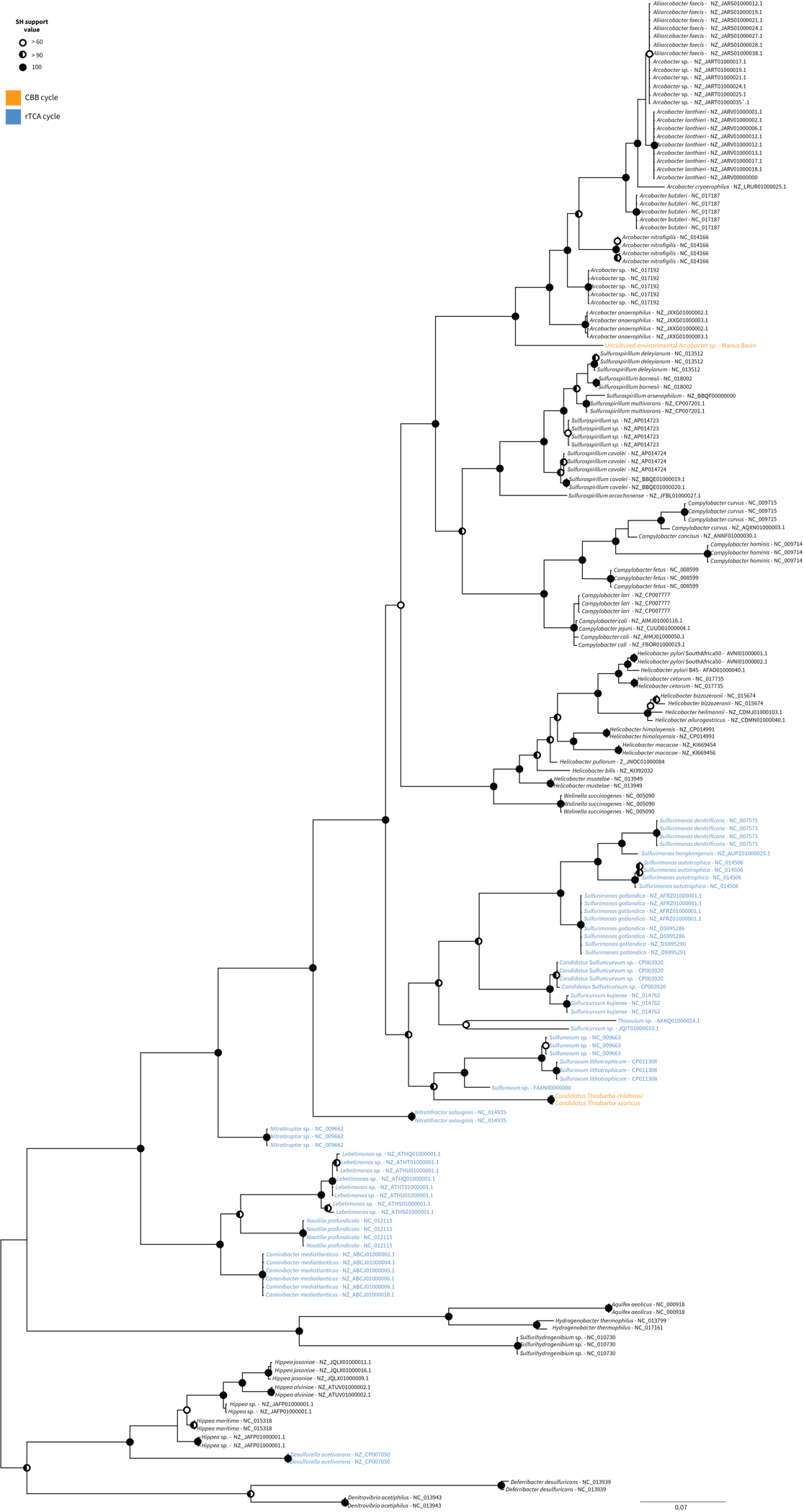


### Figure S2. 16s rRNA phylogenetic tree of representative Campylobacterota calculated using Bayesian inference. Five deltaproteobacterial species were used to root the tree. In blue are genomes with rTCA cycle genes and in orange genomes with CBB cycle genes.


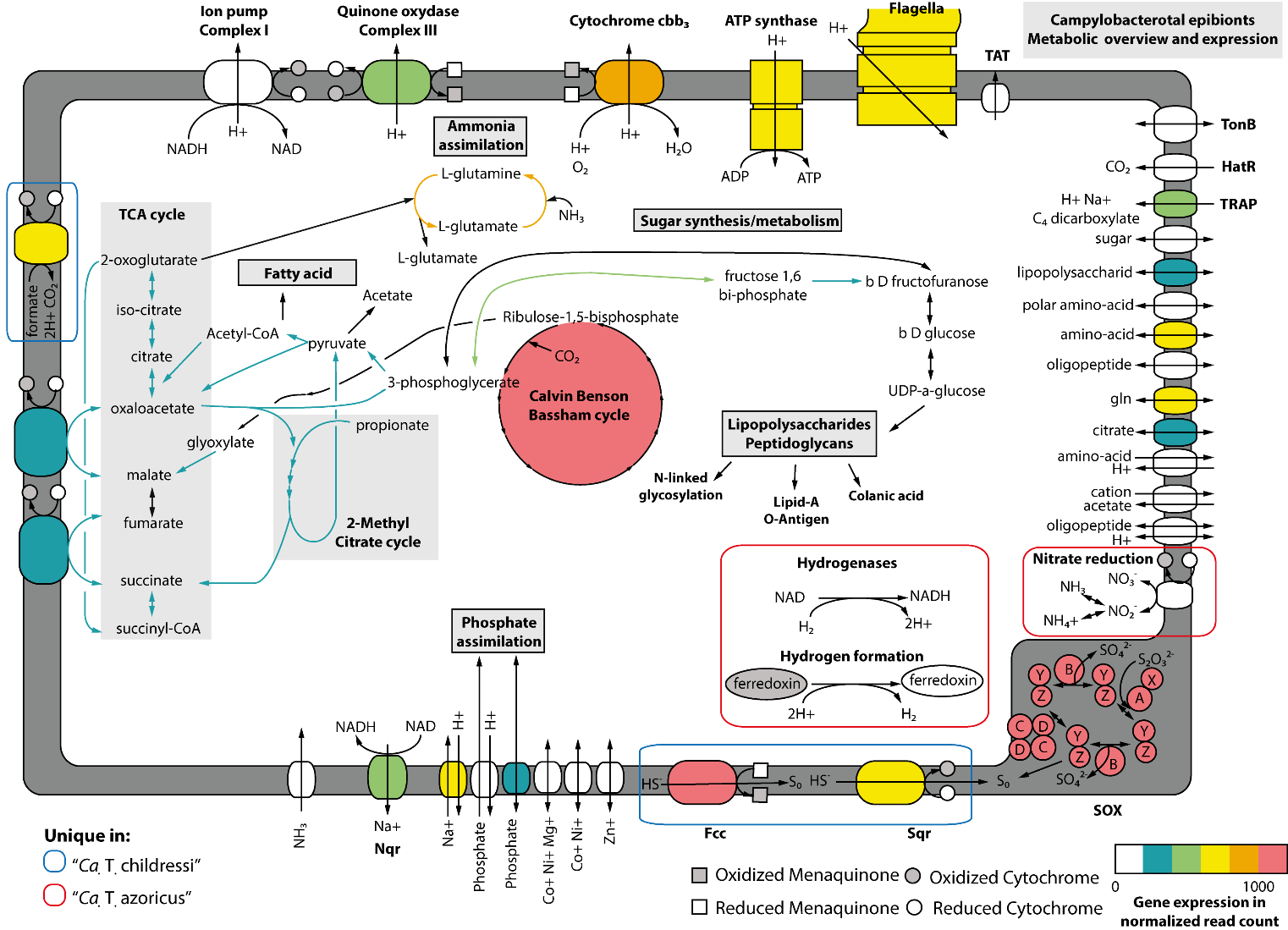


**Figure S3.** Diagram of main metabolic pathways annotated in the “*Ca*. Thiobarba” genomes. Colors show the transcription expression levels of “*Ca*. T. childressi” pathways. Framed with a red line are the metabolic reactions only found in the “*Ca*. T. azoricus” genome and with a blue line the reactions only found in “*Ca*. T. childressi”.


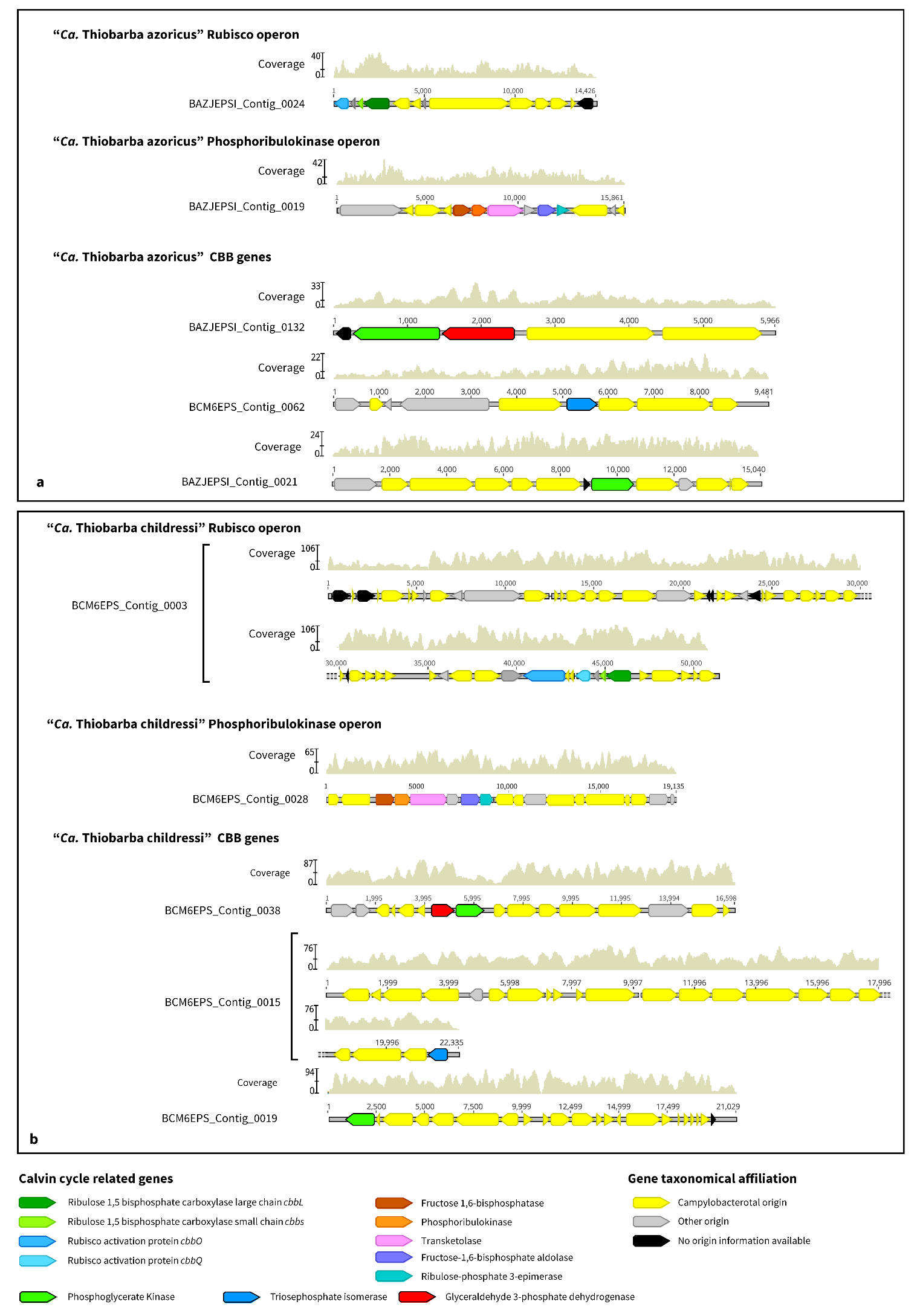


**Figure S4.** Graphic representation of the contigs containing RuBisCO and phosphoribulokinase gene clusters from “*Ca* T. childressi” (**a**) and “*Ca* T. azoricus” (**b**).


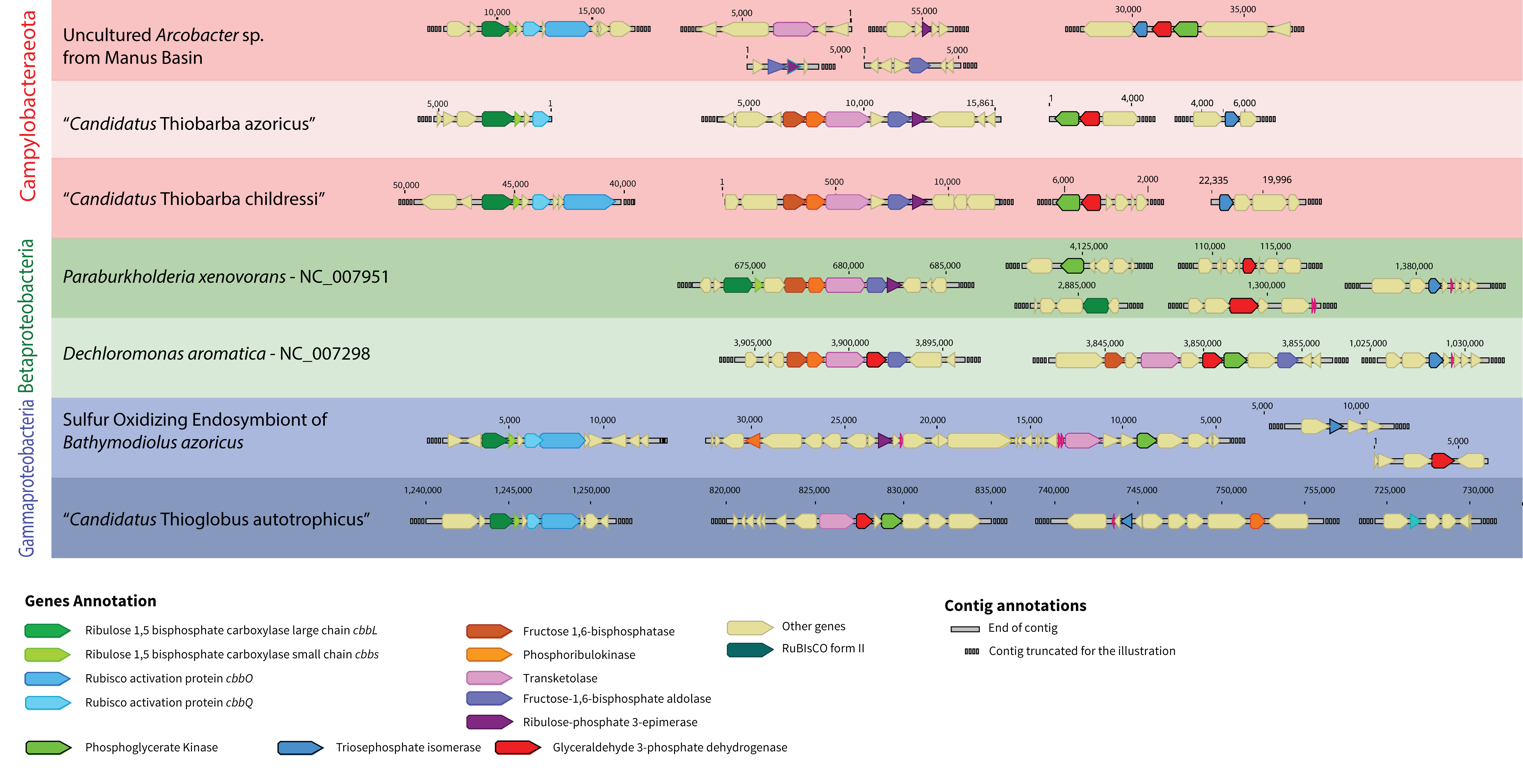


**Figure S5.** Graphic representation of the RuBisCO and phosphoribulokinase genes clusters in “*Ca*. Thiobarba” species compared to genomes with closely related gene similarities. The order of RuBisCO genes was similar to that of Gammaproteobacteria associated with bathymodiolin mussels whereas the CBB cycle accessory genes shared a similar order with genes found in Betaproteobacteria.

**
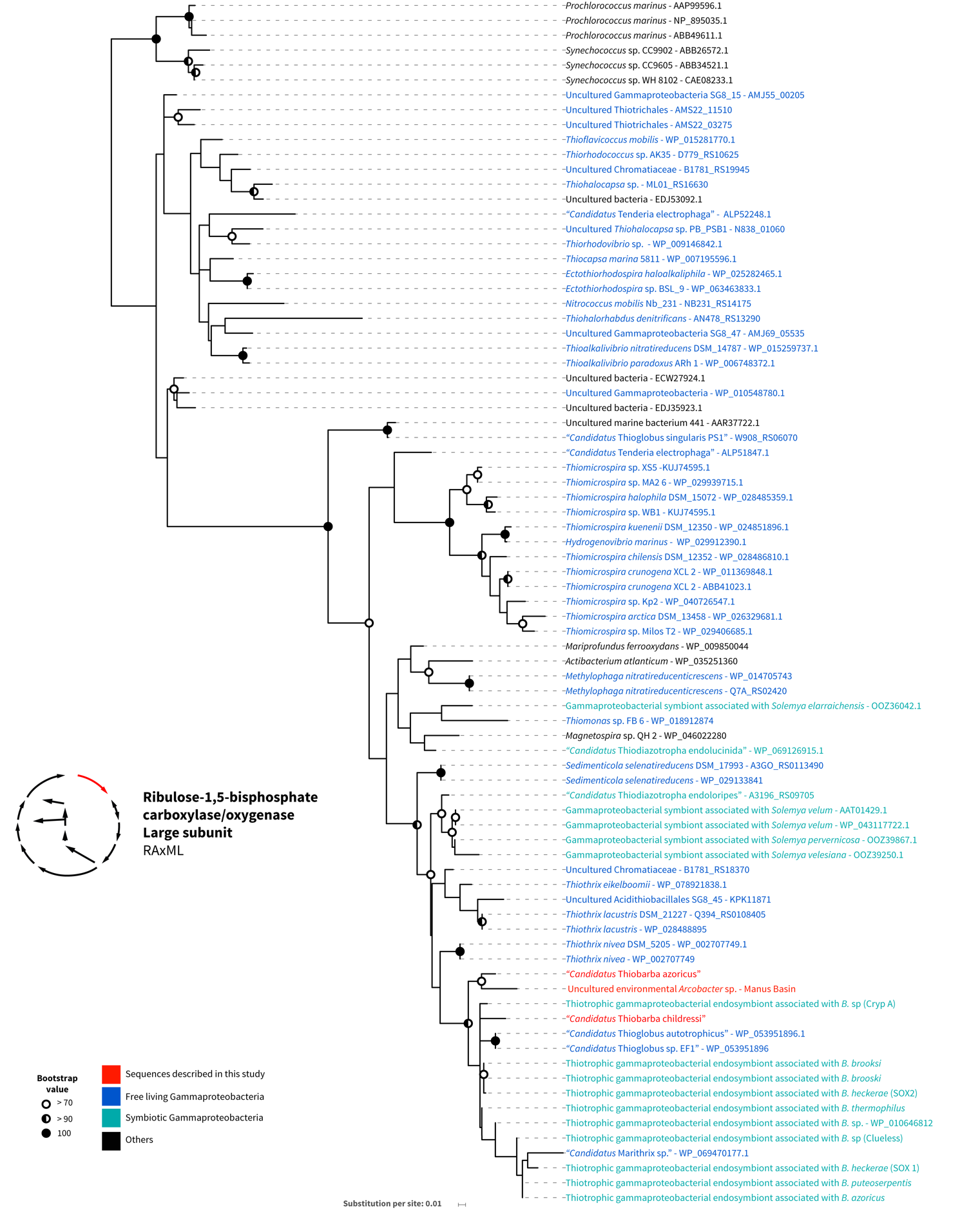
Figure S6** Maximum likelihood tree of amino acid sequences of the ribulose 1,5-bisphosphate carboxylase large subunit involved in the CBB cycle. The model used for each gene is detailed in Table S7. Analyses were performed with RAxML using 1000 bootstraps.


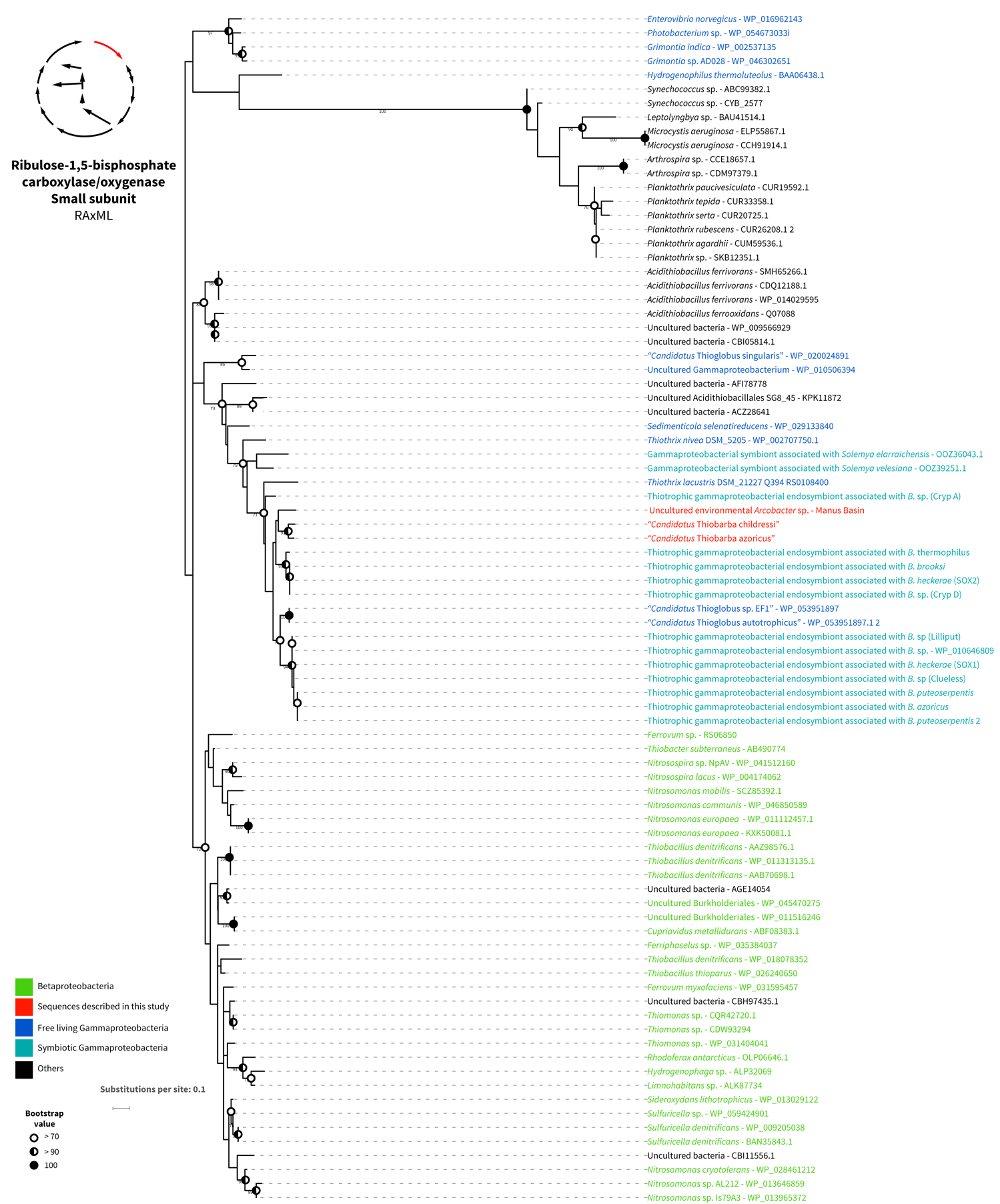


**Figure S7** Maximum likelihood tree of amino acid sequences of the ribulose 1,5-bisphosphate carboxylase small subunit involved in the CBB cycle. The model used for each gene is detailed in Table S7. Analyses were performed with RAxML using 1000 bootstraps.


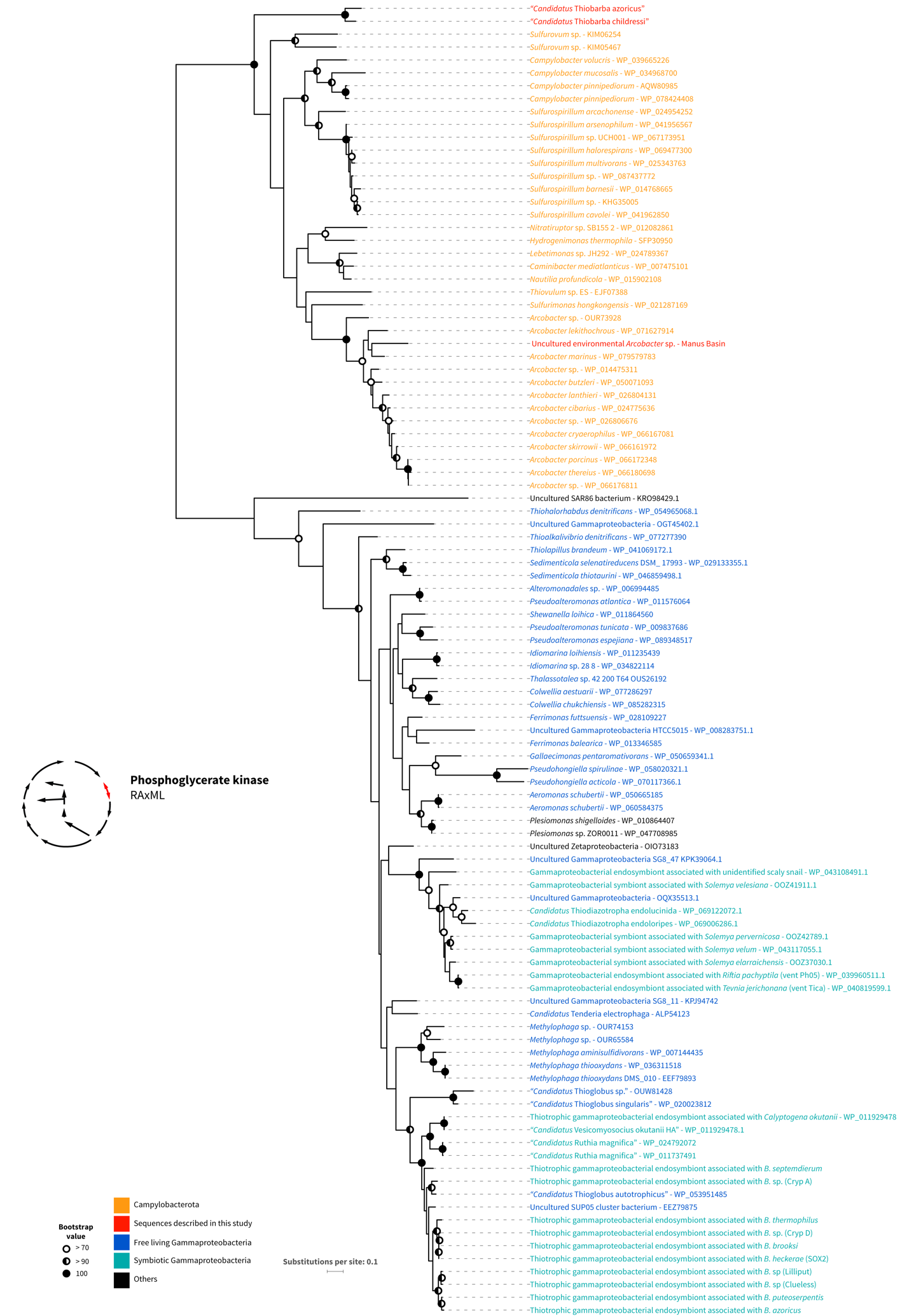


**Figure S8** Maximum likelihood tree of amino acid sequences of the phosphoglycerate kinase involved in the CBB cycle. The model used for each gene is detailed in Table S7. Analyses were performed with RAxML using 1000 bootstraps.


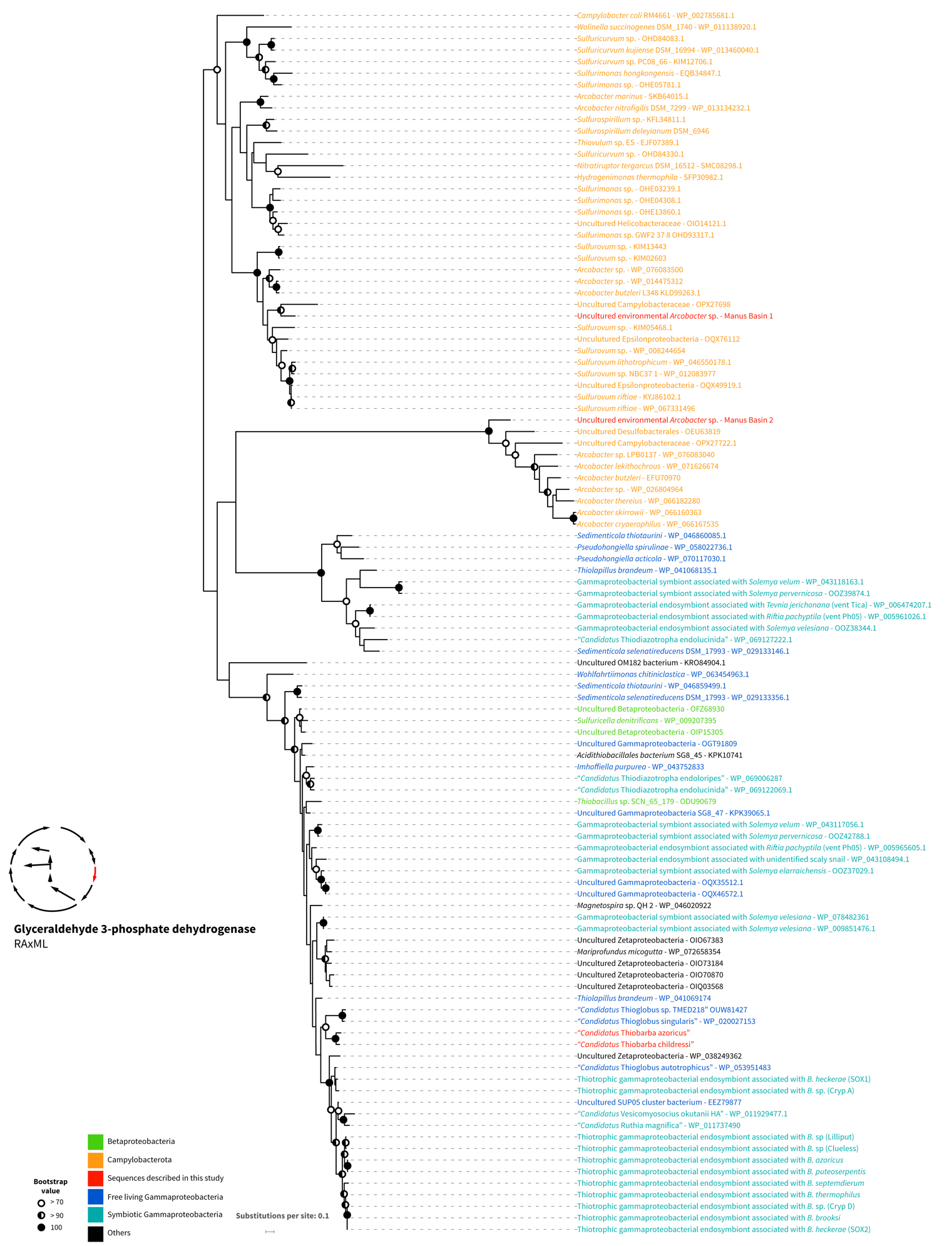


**Figure S9.** Maximum likelihood tree of amino acid sequences of the glyceraldehyde 3-phosphate dehydrogenase involved in the CBB cycle. The model used for each gene is detailed in Table S7. Analyses were performed with RAxML using 1000 bootstraps.


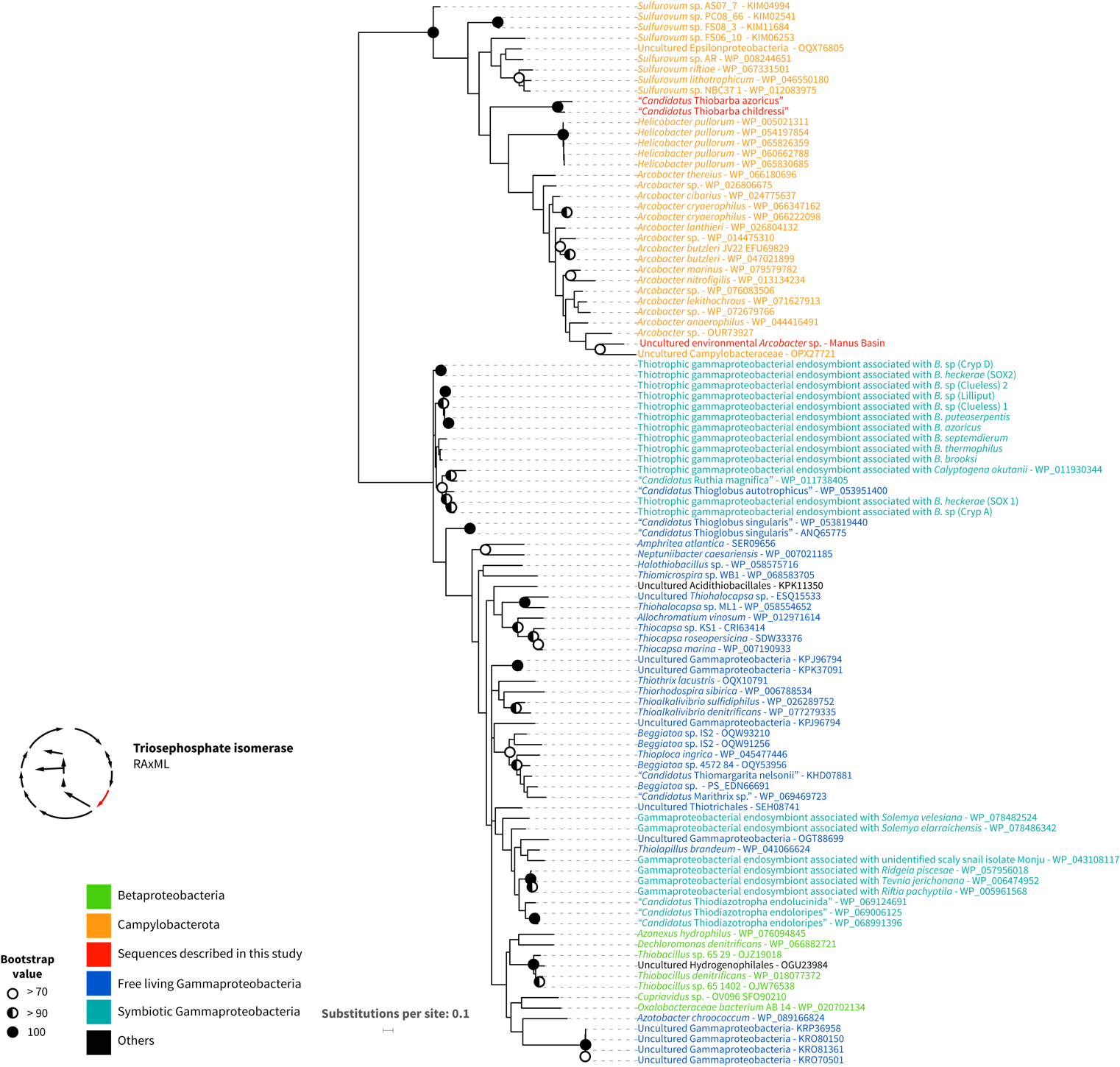


**Figure S10** Maximum likelihood tree of amino acid sequences of the triosephosphate isomerase involved in the CBB cycle. The model used for each gene is detailed in Table S7. Analyses were performed with RAxML using 1000 bootstraps.


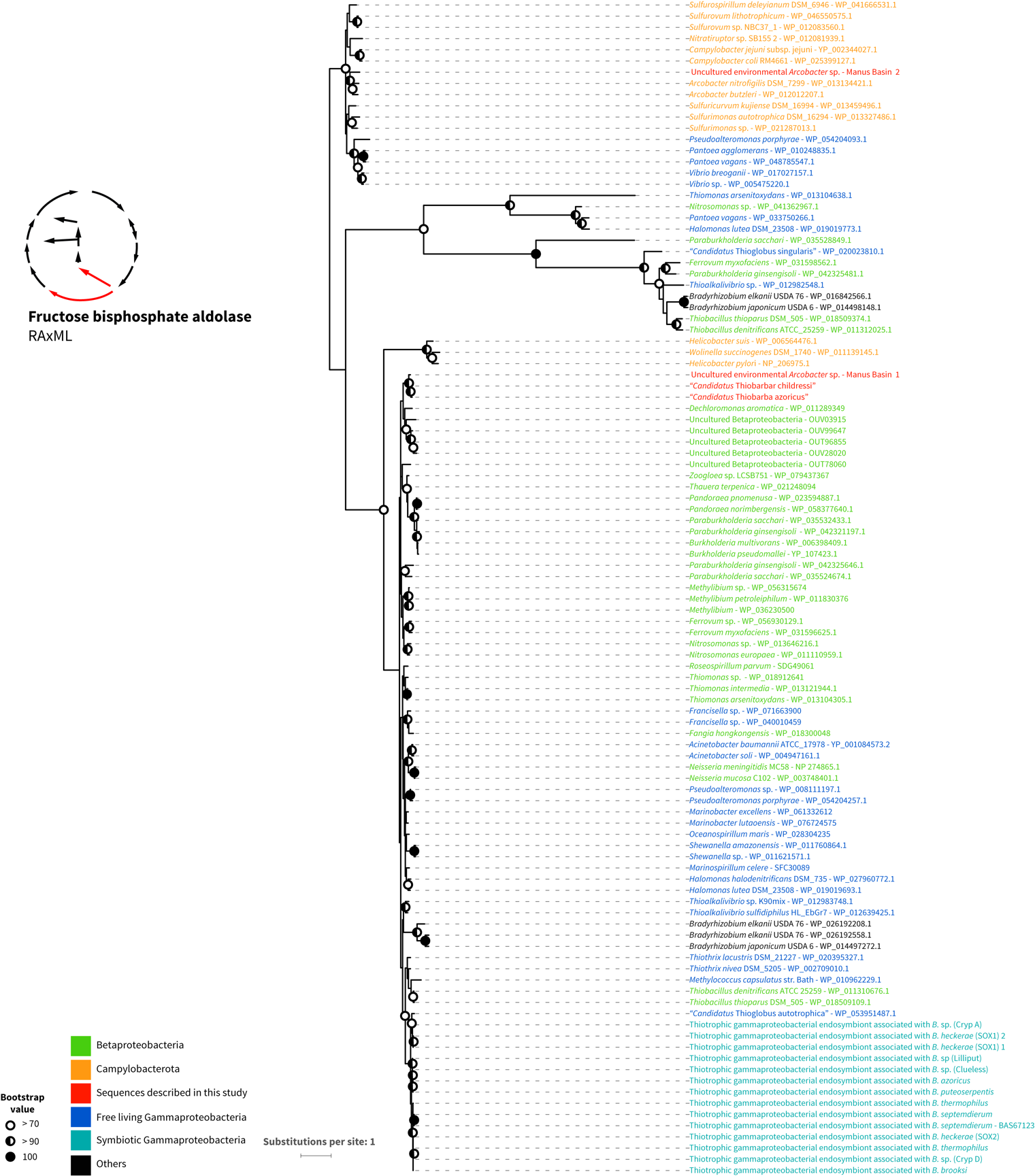


**Figure S11** Maximum likelihood tree of amino acid sequences of the fructose bisphosphate aldolase involved in the CBB cycle. The model used for each gene is detailed in Table S7. Analyses were performed with RAxML using 1000 bootstraps.


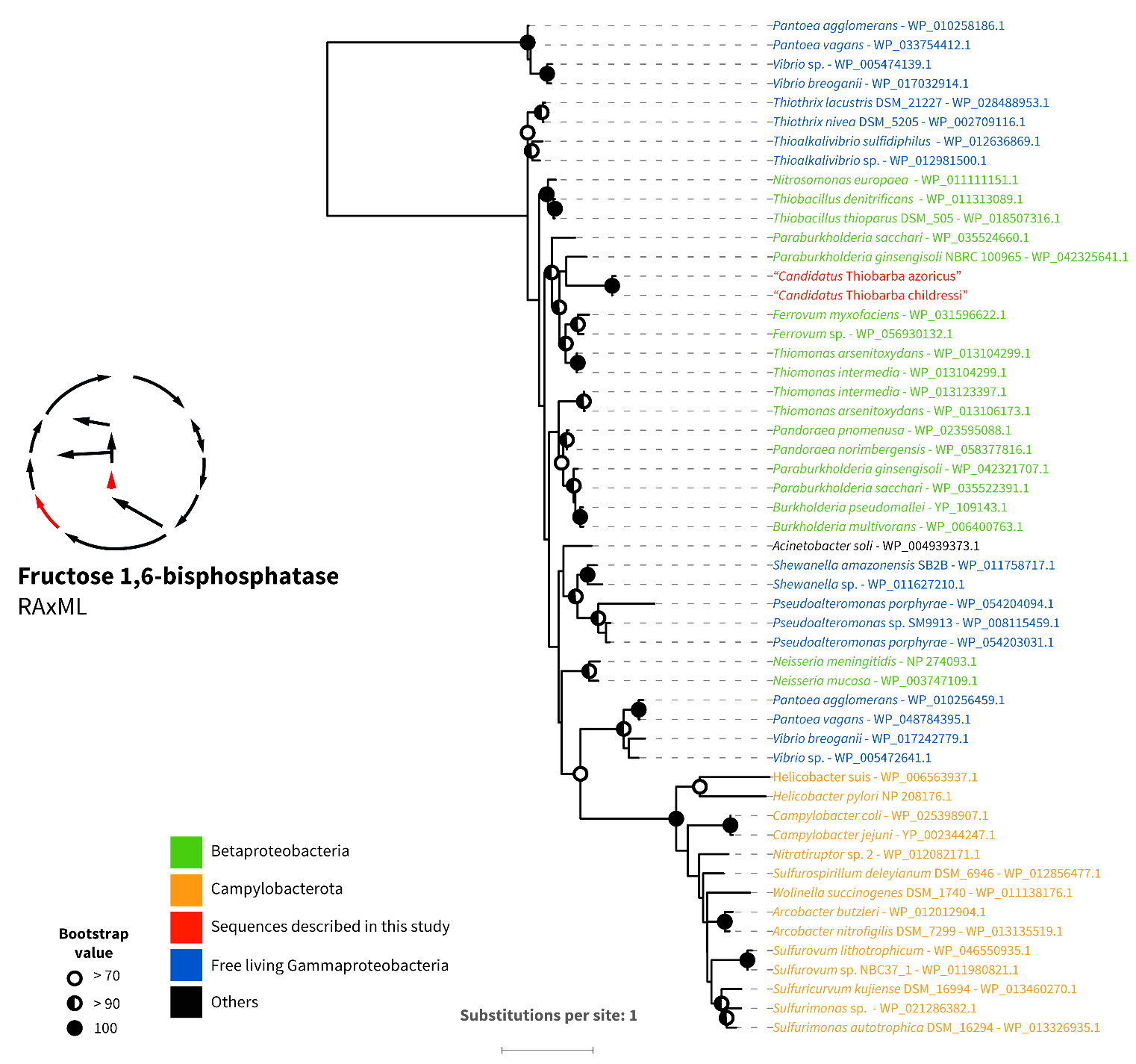


**Figure S12** Maximum likelihood tree of amino acid sequences of the fructose 1,6-bisphosphatase involved in the CBB cycle. The model used for each gene is detailed in Table S7. Analyses were performed with RAxML using 1000 bootstraps.


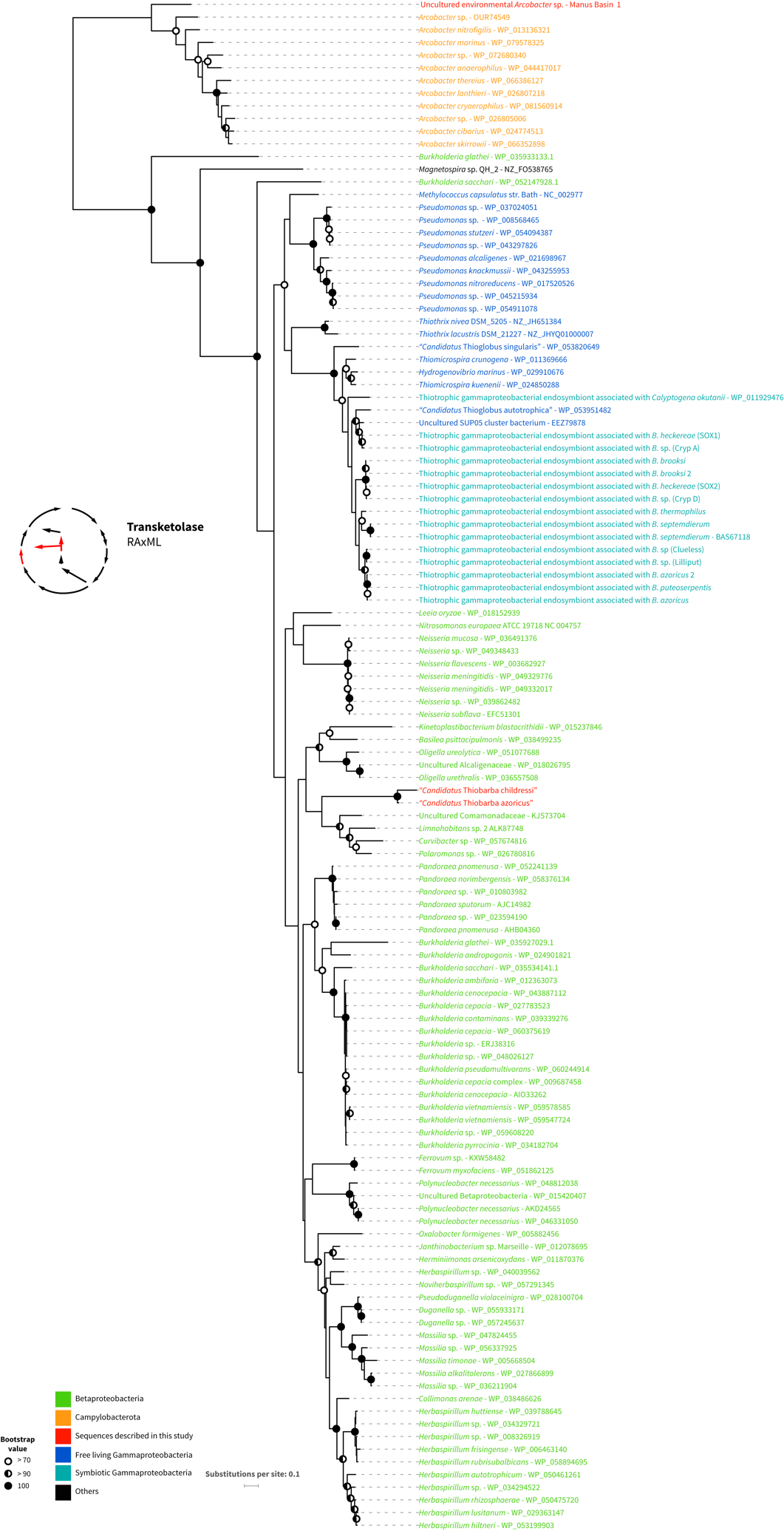


**Figure S13** Maximum likelihood tree of amino acid sequences of the transketolase involved in the CBB cycle. The model used for each gene is detailed in Table S7. Analyses were performed with RAxML using 1000 bootstraps.


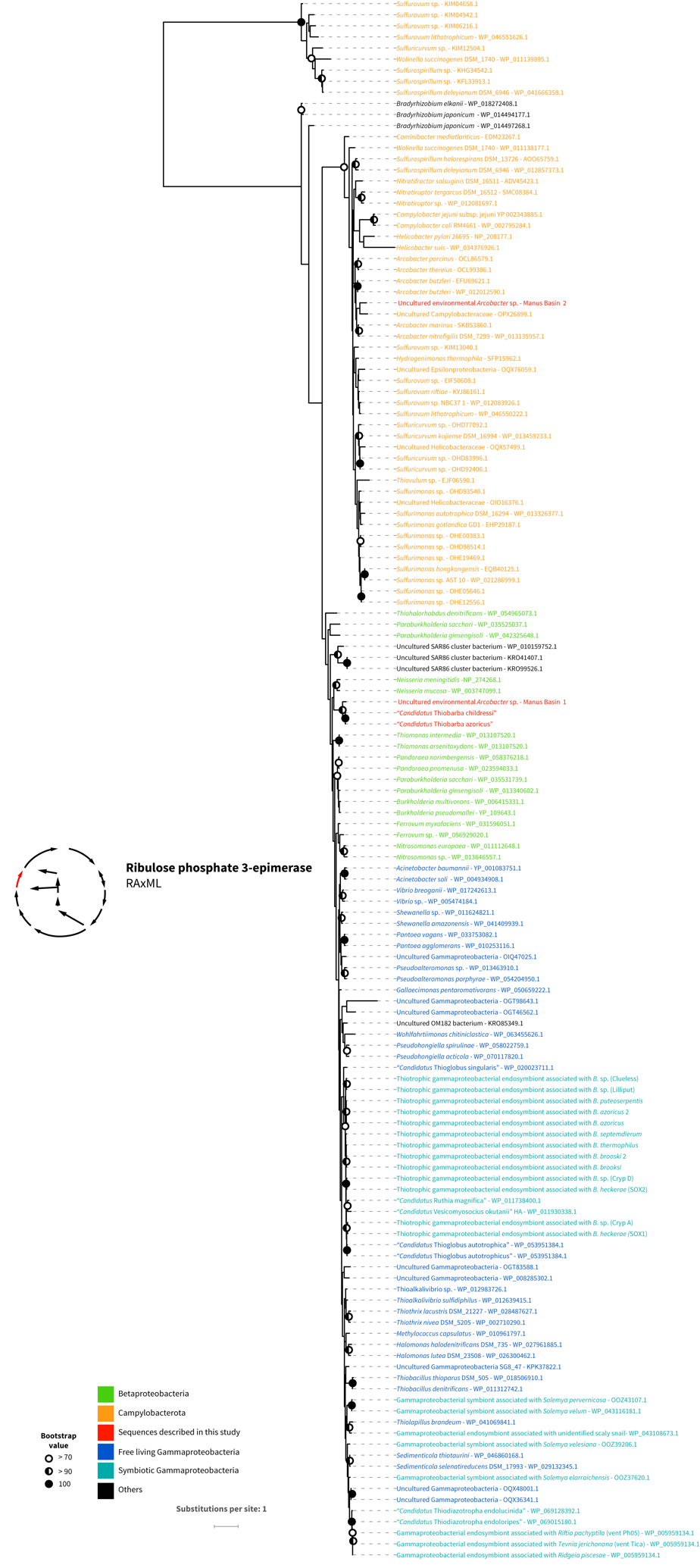


**Figure S14** Maximum likelihood tree of amino acid sequences of the ribulose-phosphate-3-epimerase involved in the CBB cycle. The model used for each gene is detailed in Table S7. Analyses were performed with RAxML using 1000 bootstraps.


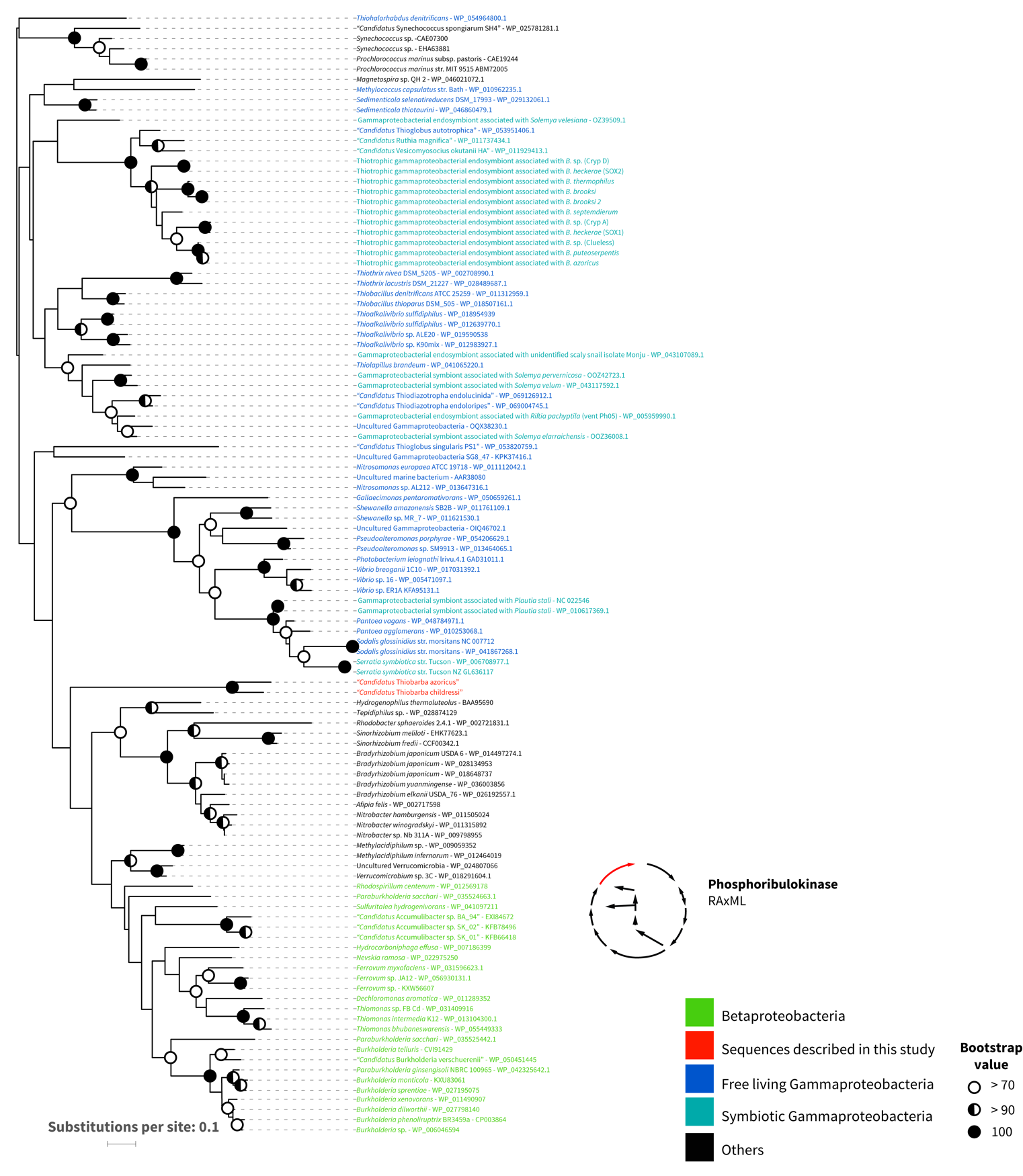


**Figure S15** Maximum likelihood tree of amino acid sequences of the phosphoribulokinase involved in the CBB cycle. The model used for each gene is detailed in Table S7. Analyses were performed with RAxML using 1000 bootstraps.


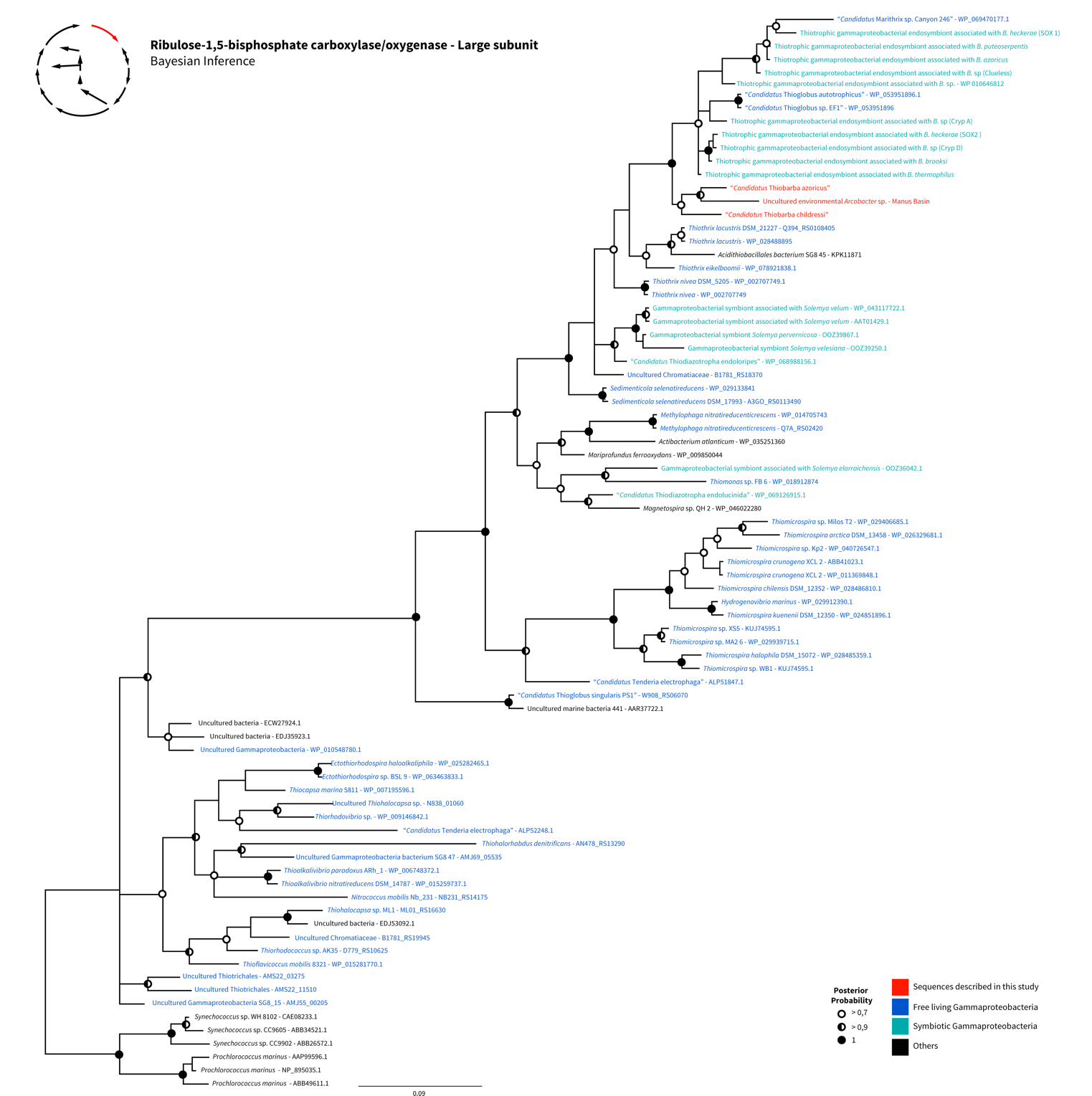


**Figure S16** Bayesian inference tree of the ribulose 1,5-bisphosphate carboxylase large subunit involved in the CBB cycle. Analyses were performed with 6 million generations using two parallel Monte Carlo Markov chains. Sample trees were taken every 25000 generations.


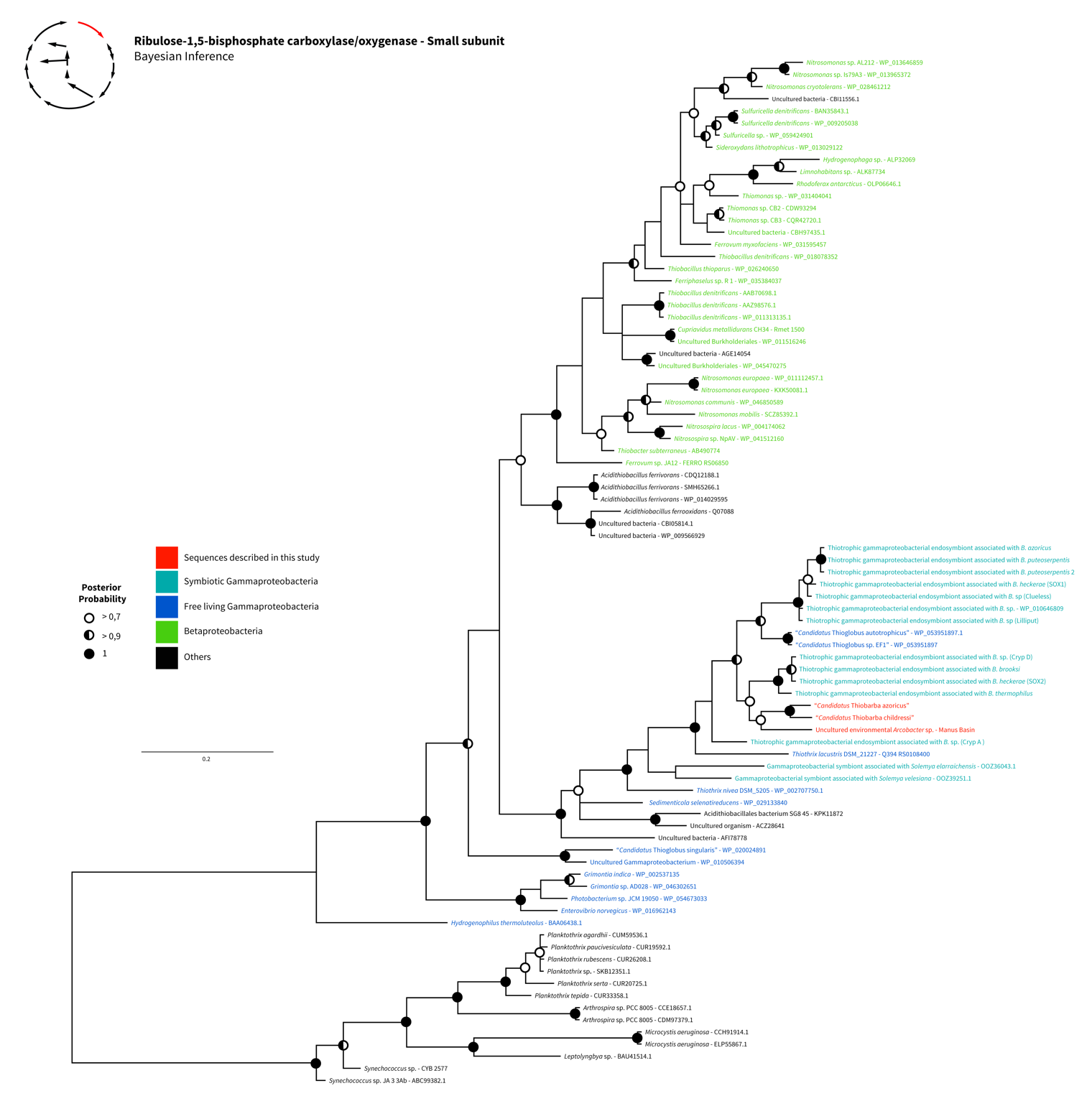


**Figure S17** Bayesian inference tree of the ribulose 1,5-bisphosphate carboxylase small subunit involved in the CBB cycle. Analyses were performed with 6 million generations using two parallel Monte Carlo Markov chains. Sample trees were taken every 25000 generations.


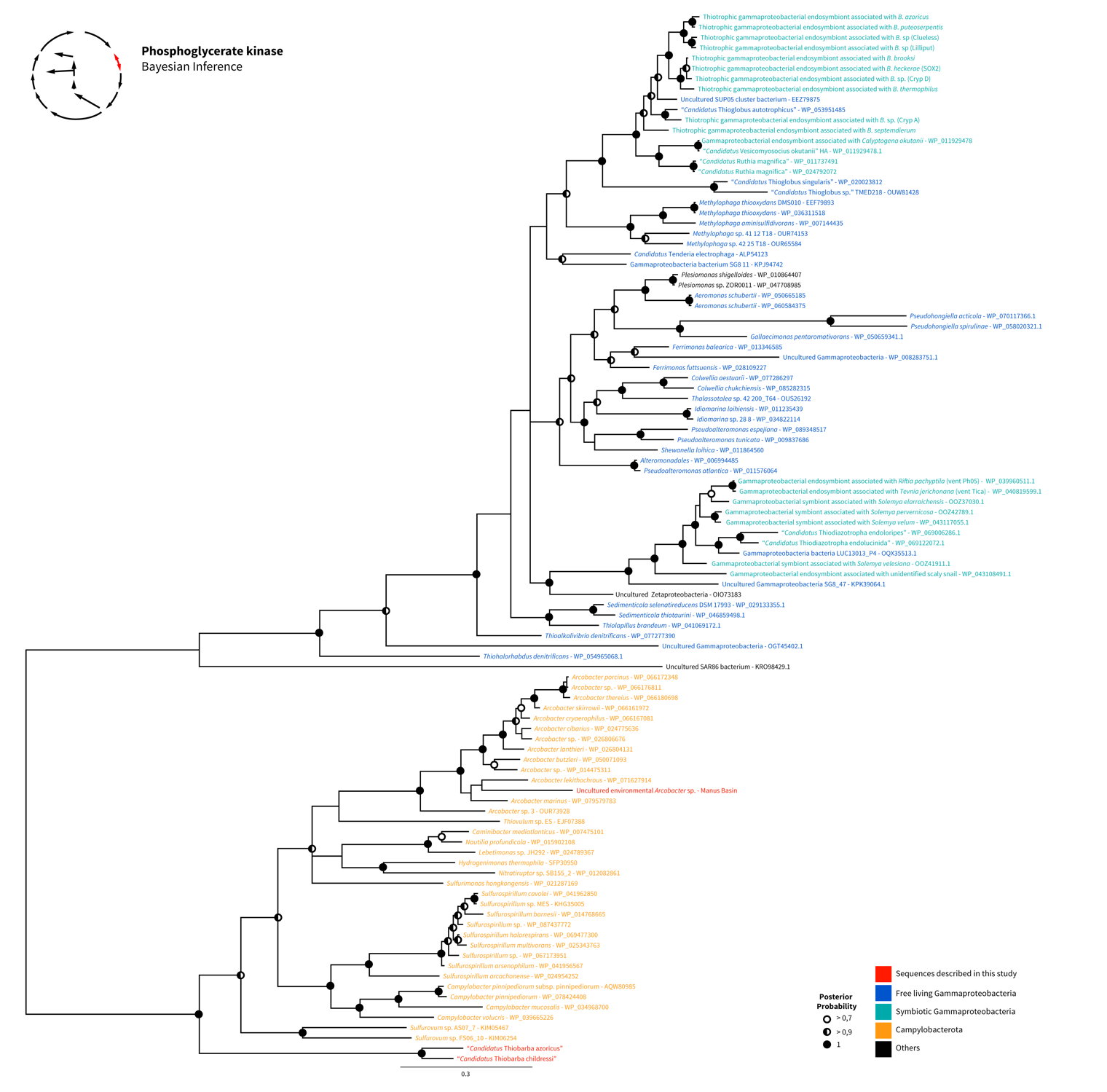


**Figure S18** Bayesian inference tree of Phosphoglycerate Kinase involved in the CBB cycle. Analyses were performed with 6 million generations using two parallel Monte Carlo Markov chains. Sample tree were taken every 25000 generations.


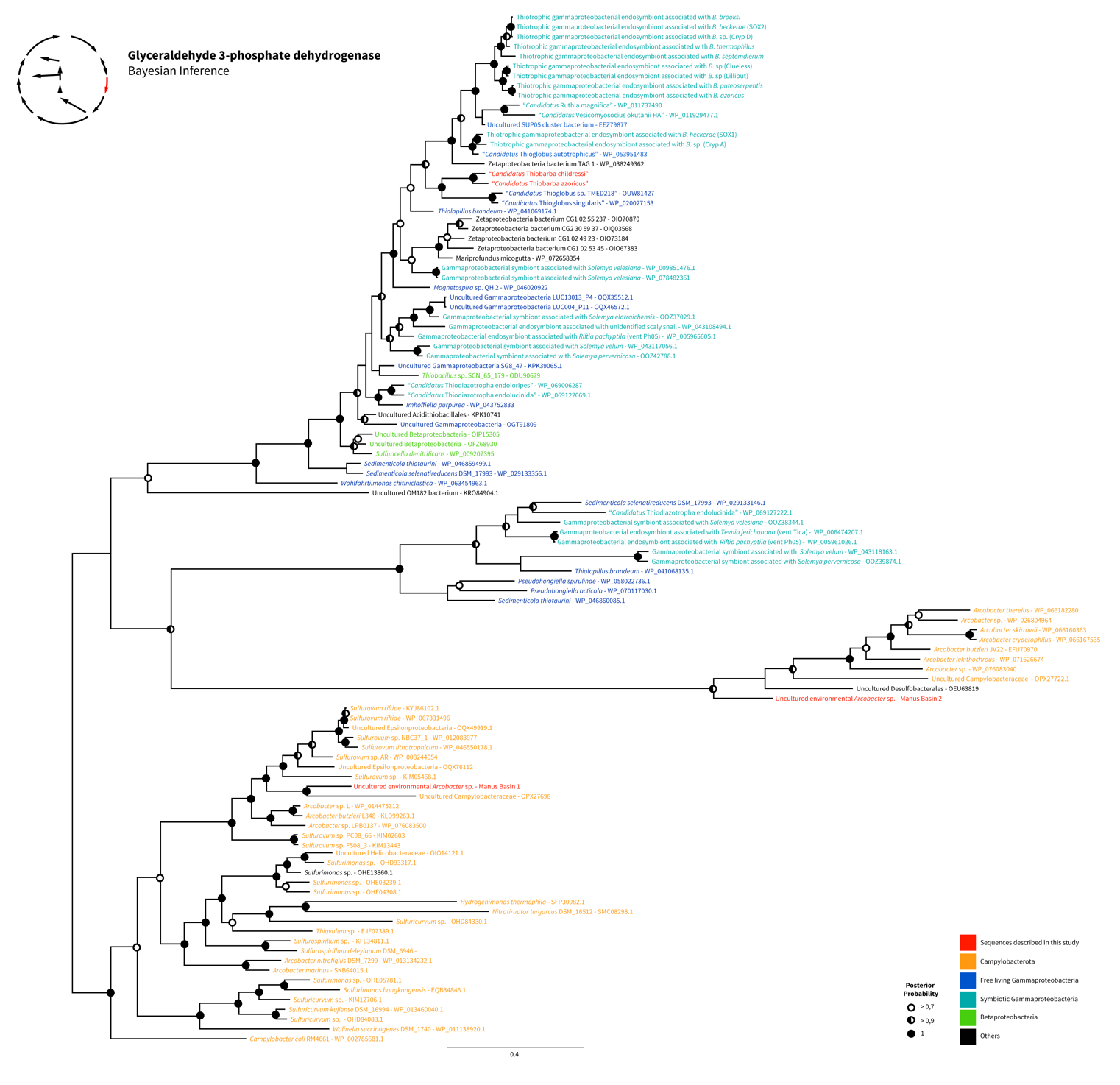


**Figure S19** Bayesian inference tree of the glyceraldehyde 3-phosphate dehydrogenase involved in the CBB cycle. Analyses were performed with 6 million generations using two parallel Monte Carlo Markov chains. Sample trees were taken every 25000 generations.


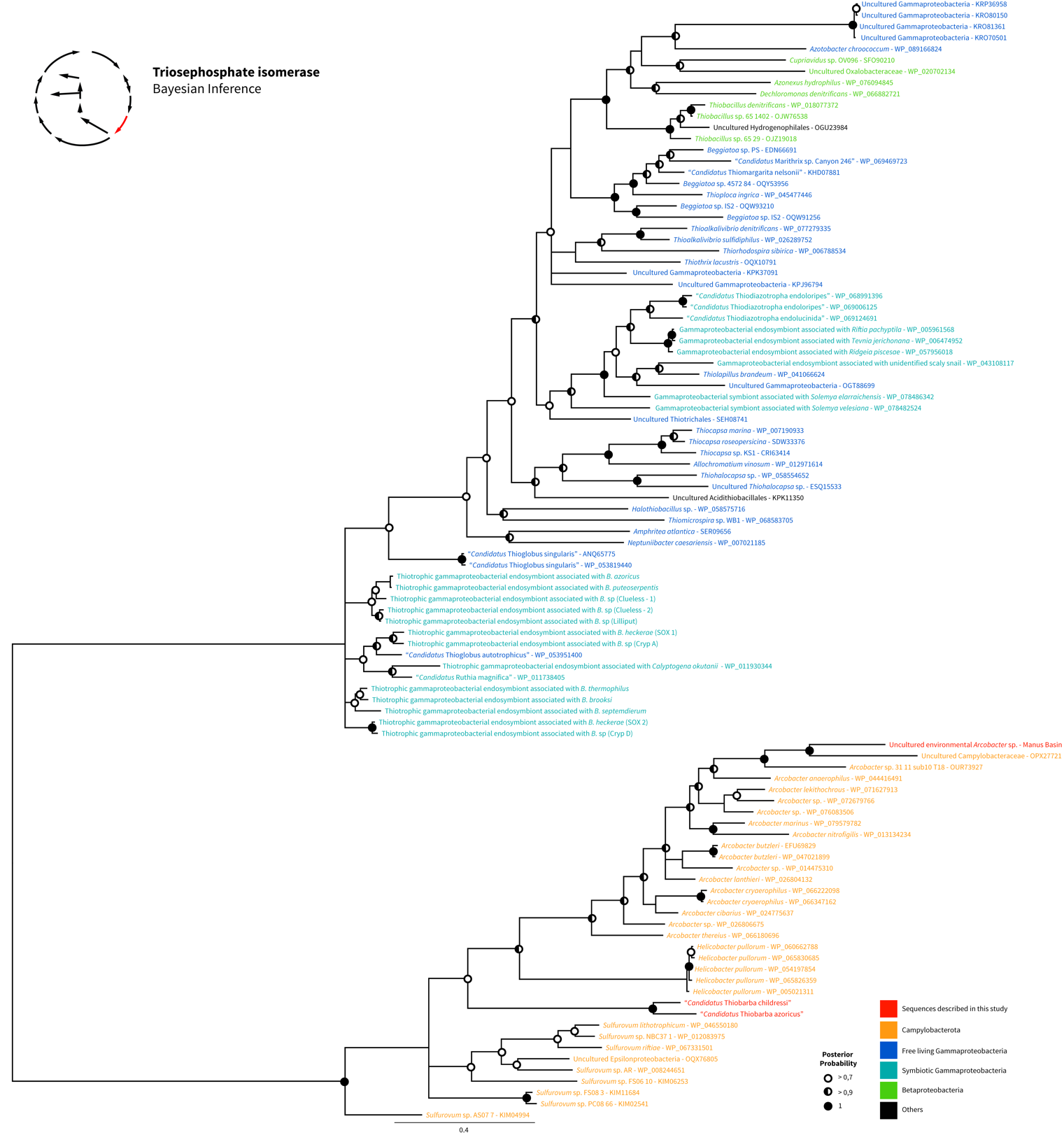


**Figure S20** Bayesian inference tree of the triosephosphate isomerase involved in the CBB cycle. Analyses were performed with 6 million generations using two parallel Monte Carlo Markov chains. Sample trees were taken every 25000 generations.


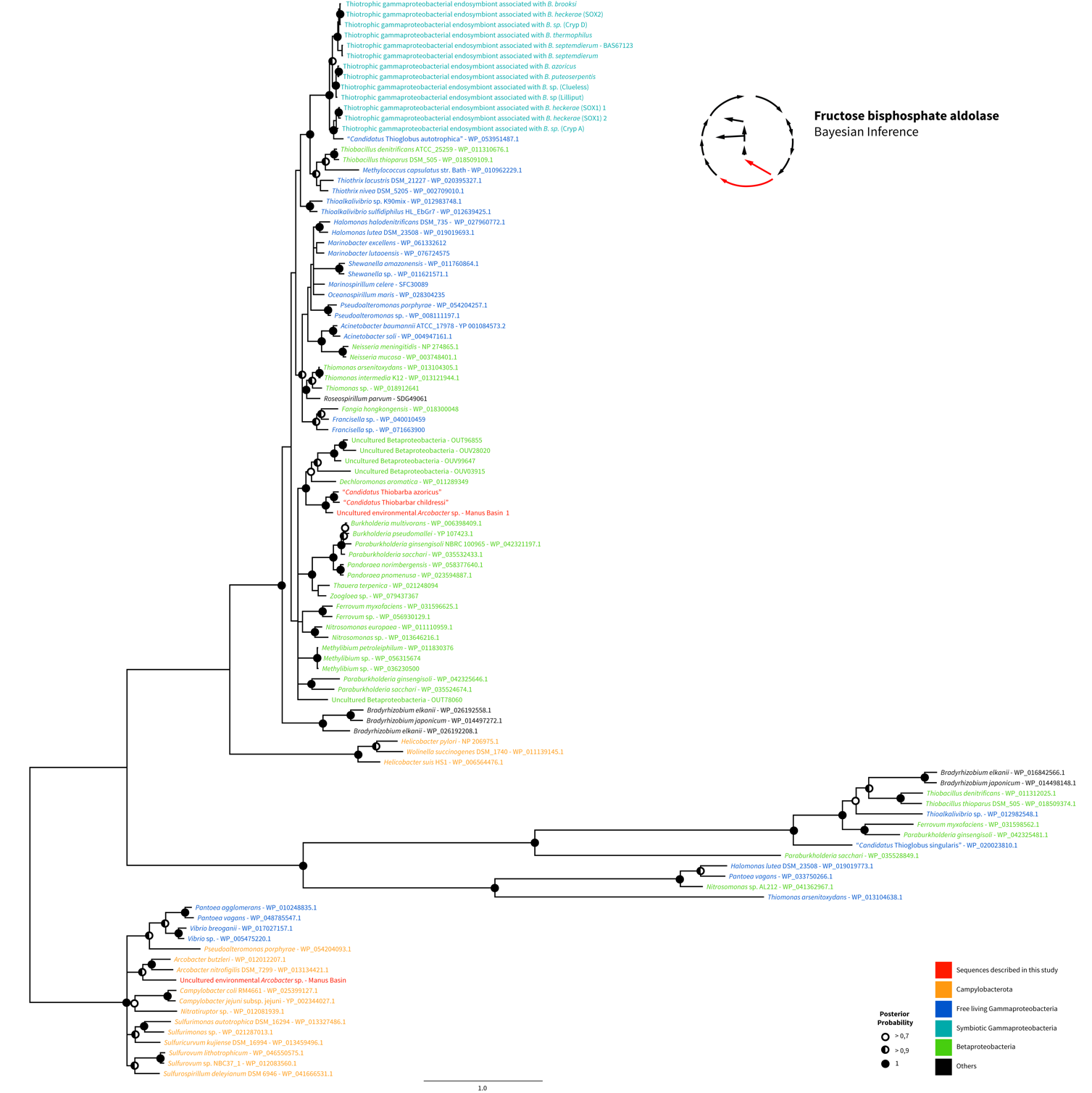


**Figure S21** Bayesian inference tree of the fructose bisphosphate aldolase involved in the CBB cycle. Analyses were performed with 6 million generations using two parallel Monte Carlo Markov chains. Sample trees were taken every 25000 generations.


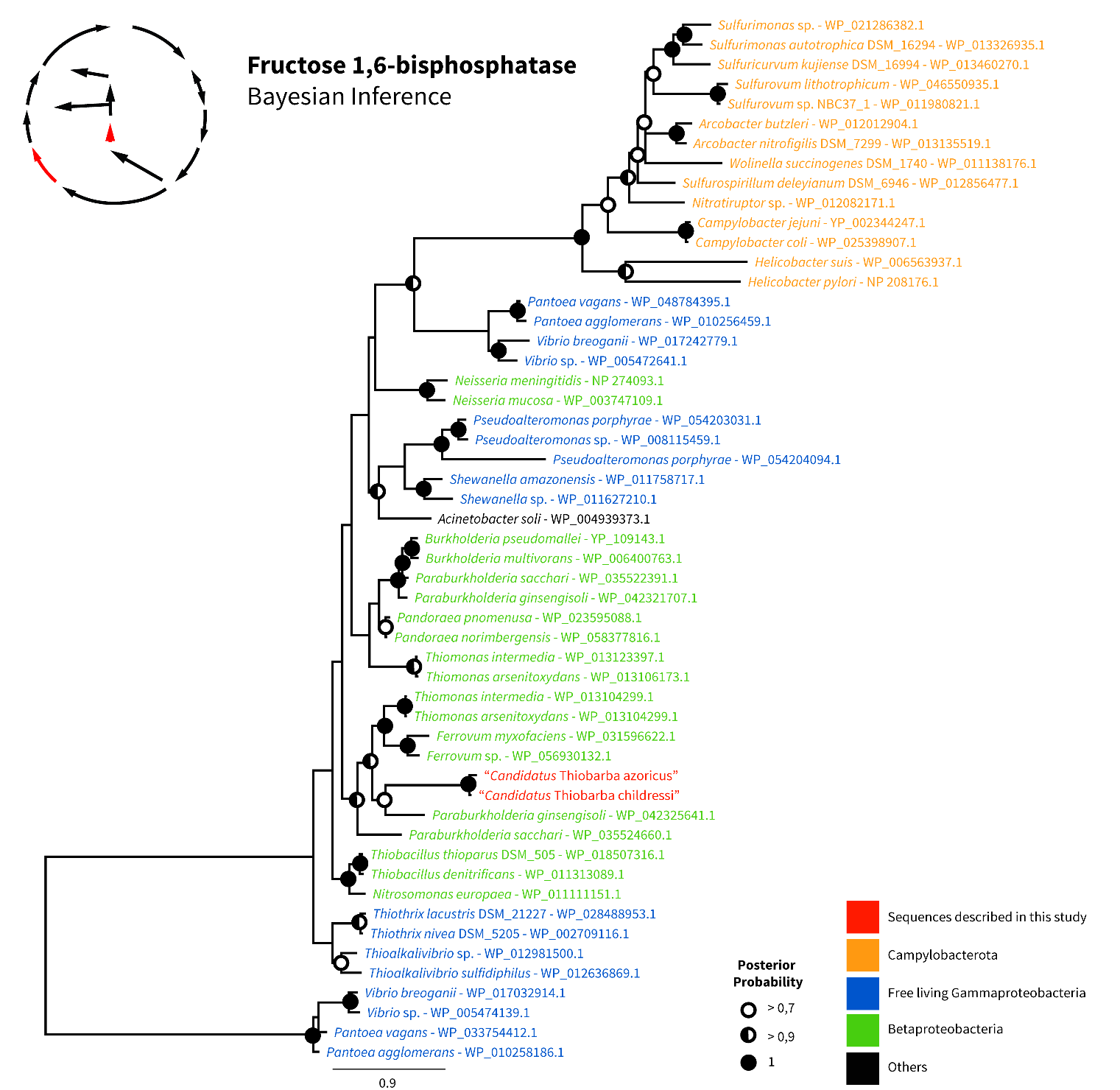


**Figure S22** Bayesian inference tree of the fructose 1,6-bisphosphatase involved in the CBB cycle. Analyses were performed with 6 million generations using two parallel Monte Carlo Markov chains. Sample trees were taken every 25000 generations.


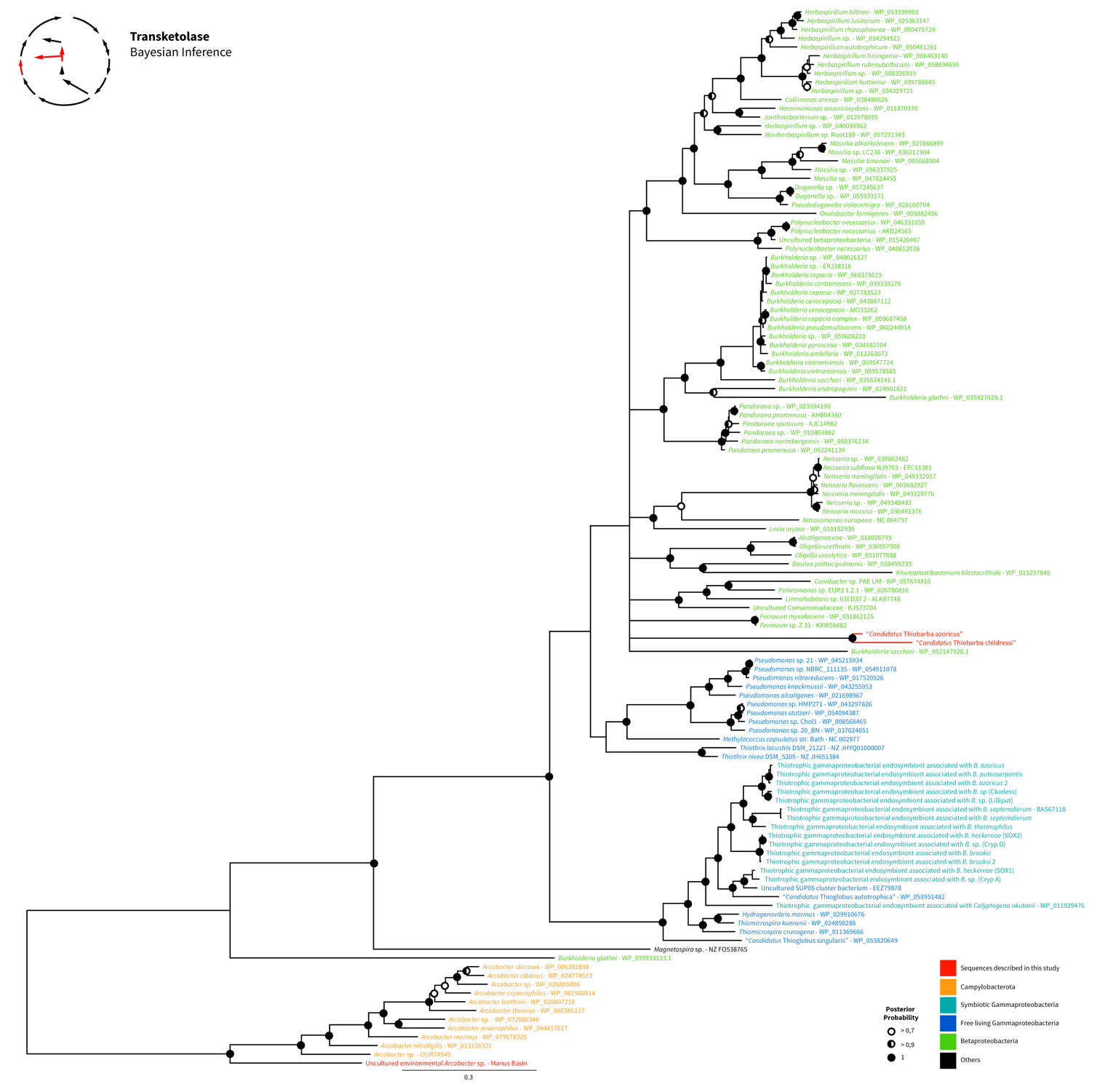


**Figure S23** Bayesian inference tree of the transketolase involved in the CBB cycle. Analyses were performed with 6 million generations using two parallel Monte Carlo Markov chains. Sample trees were taken every 25000 generations.


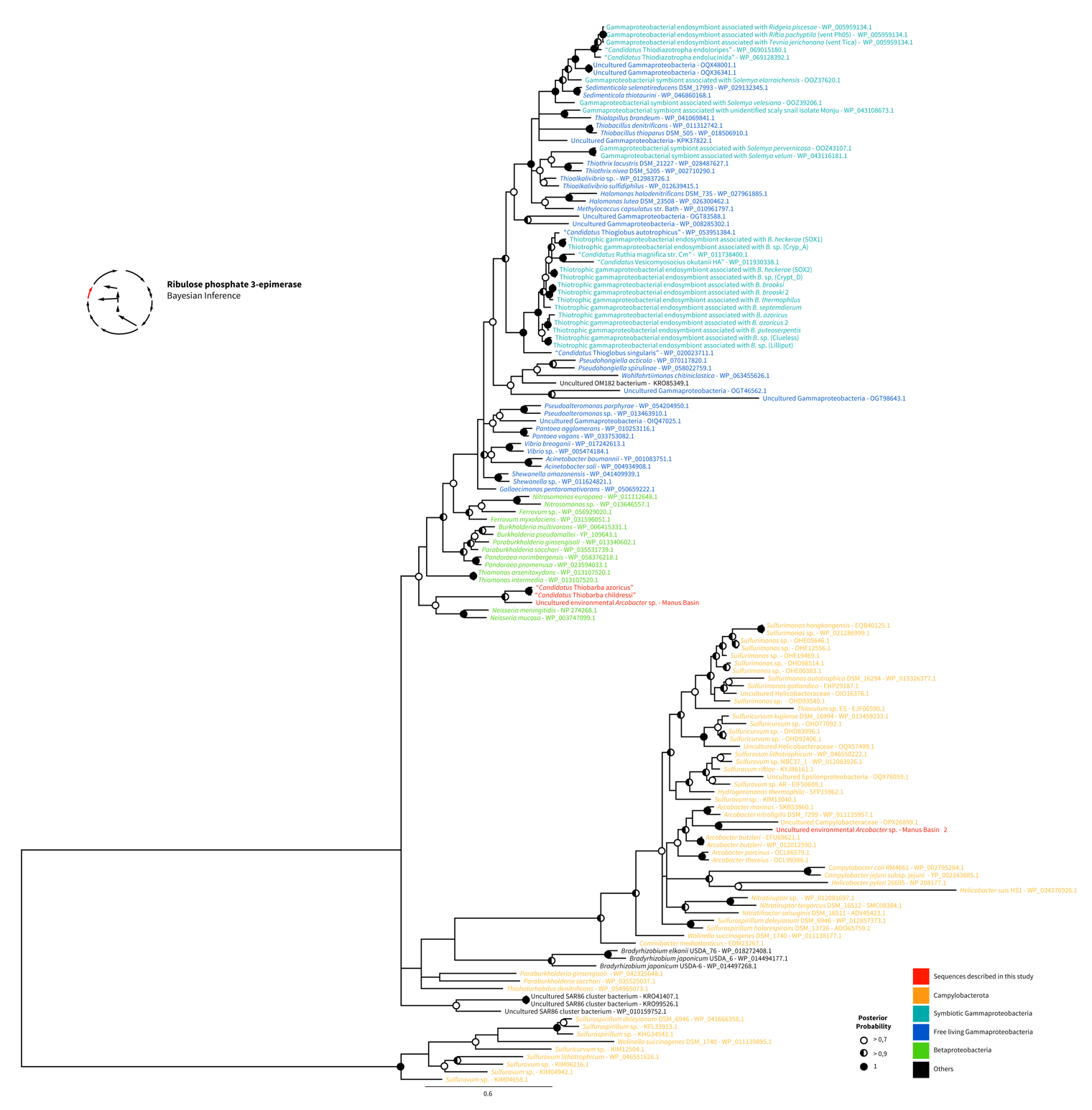


**Figure S24** Bayesian inference tree of the ribulose-phosphate-3-epimerase involved in the CBB cycle. Analyses were performed with 6 million generations using two parallel Monte Carlo Markov chains. Sample trees were taken every 25000 generations.


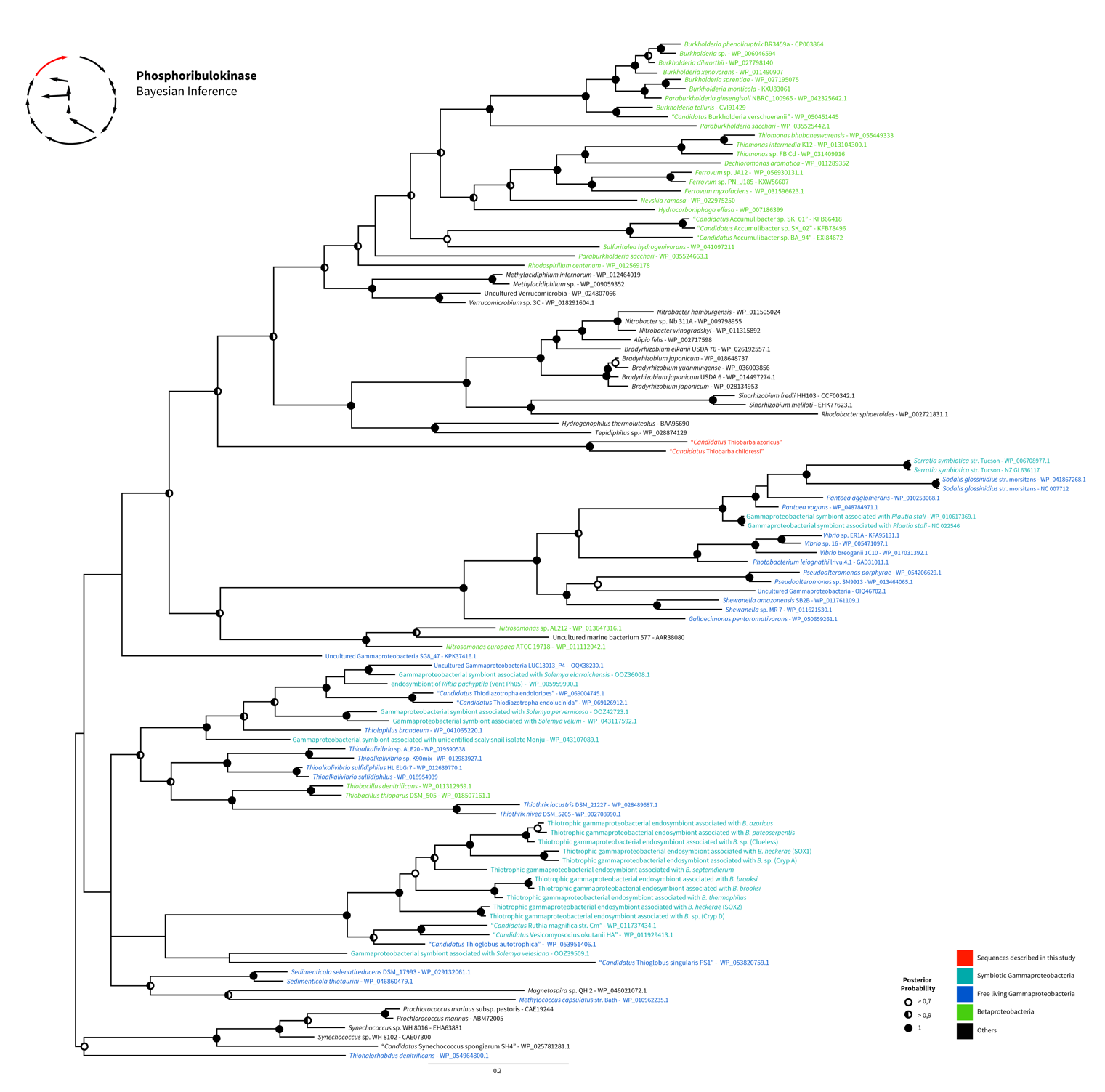


**Figure S25** Bayesian inference tree of the phosphoribulokinase involved in the CBB cycle. Analyses were performed with 6 million generations using two parallel Monte Carlo Markov chains. Sample trees were taken every 25000 generations**.**

**
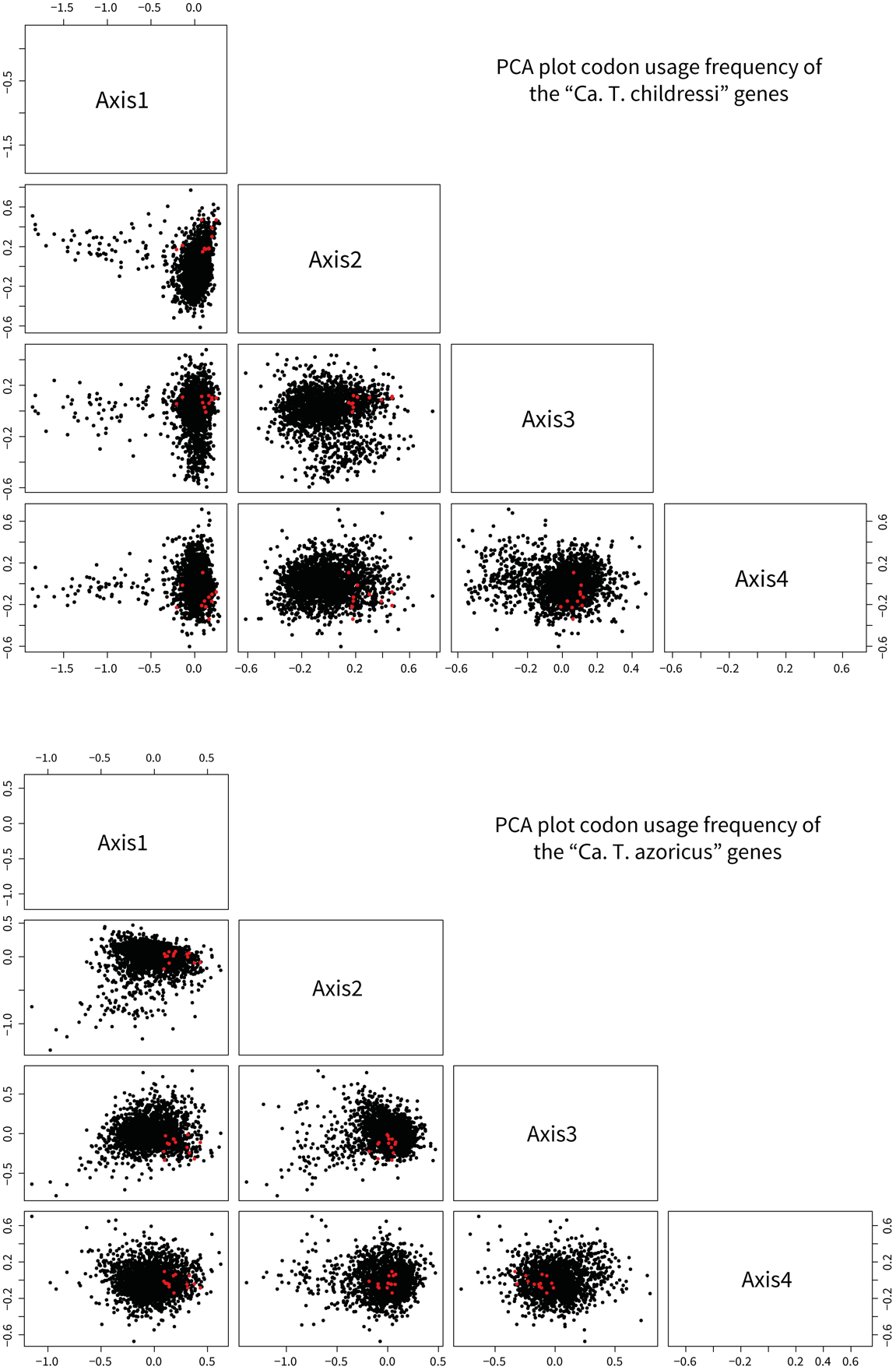
**

**Figure S26** PCA plots of codon usage in the “*Ca*. Thiobarba” genomes. Each dot represents the codon usage of one annotated gene in the “*Ca*. Thiobarba” genome, in red are the genes coding for the CBB cycle enzymes.


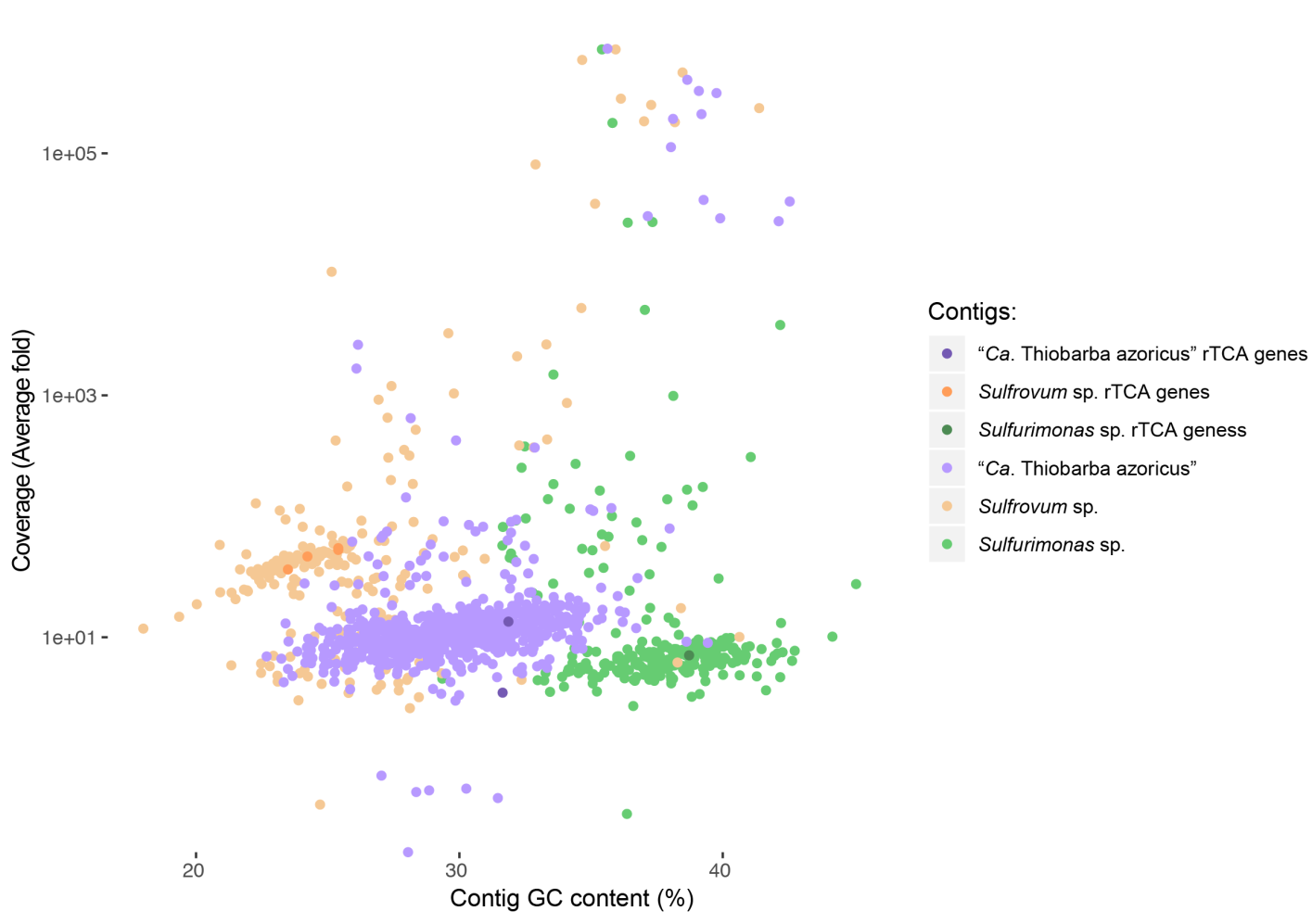


**Figure S27** Plot of coverage and GC content for each Campylobacteroa contig present in the *B. azoricus* metagenome. The contigs that encode rTCA genes are shown in darker colors. The coverage and GC content of these contigs matches the typical coverage and GC content for the bins they were assigned to.

### Supplementary information tables

The following tables are supplied as an additional Excel files:

**Table S1:** Table of genes of interest present in “*Ca.* Thiobarba”

**Table S2:** Table summarizing the average amino acids identities values between “*Ca.* Thiobarba” and other representative Campylobacterota

**Table S3:** Table ranking counts of transcriptome reads mapping to “*Ca.* Thiobarba” genes.

**Table S4:** Table ranking counts of protein mapping to “*Ca.* Thiobarba” genes.

**Table S5:** Table summarizing bulk IRMS ^13^C and ^15^N measurements

**Table S6:** Table with BLAST results for genes involved in the CBB cycle in “*Ca.* Thiobarba”

**Table S7:** Table summarizing metadata of samples used in this study.

**Table S8:** Table summarizing models used for the phylogenetic reconstruction of CBB genes.

Table S9: Table summarizing the Campylobacteroa genomes used in the rTCA genes mapping operation

### Supplementary References

1. Chen Z W et al. (1994) The structure of flavocytochrome c sulfide dehydrogenase from a purple phototrophic bacterium. *Science* 266, 430–432.

2. Friedrich C G, Rother D, Bardischewsky F, Ouentmeier A, Fischer J (2001) Oxidation of reduced inorganic sulfur compounds by bacteria: emergence of a common mechanism? *Appl. Environ. Microbiol.* 67, 2873–2882.

3. Brito J A et al. (2009) Structural and functional insights into sulfide:quinone oxidoreductase. *Biochemistry* 48, 5613–5622.

4. Forte E, Giuffrè A. (2016). How bacteria breathe in hydrogen sulphide-rich environments. *The Biochemist*, 38(5), 8–11.

5. Jacobi A, Rossmann R, Böck A. (1992) The hyp operon gene products are required for the maturation of catalytically active hydrogenase isoenzymes in *Escherichia* *coli*. *Arch. Microbiol.* 158, 444–451.

6. Vignais P M, Colbeau, A (2004) Molecular biology of microbial hydrogenases. *Curr. Issues Mol. Biol.* 6, 159–188.

7. Petersen J M et al. (2011) Hydrogen is an energy source for hydrothermal vent symbioses. *Nature* 476, 176–180.

8. Abaibou H et al. (1995) Expression and characterization of the *Escherichia* *coli* fdo locus and a possible physiological role for aerobic formate dehydrogenase. *J. Bacteriol.* 177(24), 7141–7149.

9. Iwadate Y et al. (2017) Involvement of formate dehydrogenases in stationary phase oxidative stress tolerance in Escherichia coli. *FEMS Microbiol. Lett,* 364, 20.

10. Markert S et al. (2007) Physiological proteomics of the uncultured endosymbiont of *Riftia* *pachyptila*. *Science (80).* 315, 247–250.

11. Ponsard J et al. (2013). Inorganic carbon fixation by chemosynthetic ectosymbionts and nutritional transfers to the hydrothermal vent host-shrimp *Rimicaris* *exoculata*. *ISME J.* 7, 96–109

12. Nakagawa S, Takaki Y. Nonpathogenic Epsilonproteobacteria. *Encyclopedia of Life Sciences*. 2009. John Wiley & Sons, Ltd, Chichester, UK, pp 1–11.

13. Nakagawa S, Takaki Y, Shimamura S, Reysenbach A-L, Takai K, Horikoshi K. Deep-sea vent -proteobacterial genomes provide insights into emergence of pathogens. *Proc Natl Acad Sci* 2007; **104**: 12146–12150.

14. Hua Q, Yang C, Oshima T, Mori H, Shimizu K (2004) Analysis of gene expression in *Escherichia* *coli* in response to changes of growth-limiting nutrient in chemostat Cultures. *Society* 70, 2354–2366.

15. Higgins C F (1992) ABC Transporters: from microorganisms to Man. *Annu. Rev. Cell Biol.* 8, 67–113.

16. Mulligan C, Fischer M, Thomas G H (2011) Tripartite ATP-independent periplasmic (TRAP) transporters in bacteria and archaea. *FEMS Microbiol. Rev.* 35, 68–86.

17. Steuber J, Krebs W. and Dimroth P (1997) The Na+‐Translocating NADH:Ubiquinone Oxidoreductase from *Vibrio alginolyticus*. Eur. J. Biochem, 249: 770-776.

18. Tanaka T, Tateno Y, Gojobori T (2005) Evolution of vitamin B6 (Pyridoxine) metabolism by gain and loss of genes. *Mol. Biol. Evol.* 22, 243–250.

19. Webb M E, Marquet A, Mendel R R, Rébeillé F, Smith A G. (2007) Elucidating biosynthetic pathways for vitamins and cofactors. *Nat. Prod. Rep.* 24, 988.

20. Stock A M, Robinson, V L, Goudreau P N (2000) Two-component signal transduction. *Annu. Rev. Biochem.* 69, 183–215.

21. Ryjenkov D A, Tarutina M, Moskvin O V, Gomelsky M. (2005) Cyclic diguanylate is a ubiquitous signaling molecule in bacteria: insights into biochemistry of the GGDEF protein domain. *J. Bacteriol.* 187, 1792–1798.

22. Nakagawa S. et al. (2007). Deep-sea vent ε-proteobacterial genomes provide insights into emergence of pathogens. *Proc. Natl. Acad. Sci.* 104, 12146–12150.

23. McSweegan E, Walker R I (1986) Identification and characterization of two *Campylobacter* *jejuni* adhesins for cellular and mucous substrates. *Infect. Immun.* 53, 141–8.

24. Gilbreath J J, Cody W L, Merrell D S, Hendrixson, D. R. (2011) Change is good: variations in common biological mechanisms in the epsilonproteobacterial genera *Campylobacter* and *Helicobacter*. *Microbiol. Mol. Biol. Rev.* 75, 84–132.

25. van der Woude M W, Baumler A J. (2004) Phase and Antigenic Variation in Bacteria. *Clin. Microbiol. Rev.* 17, 581–611.

26. Foynes S et al. (2000) *Helicobacter* *pylori* possesses two CheY response regulators and a histidine kinase sensor, CheA, which are essential for chemotaxis and colonization of the gastric mucosa. *Infect. Immun.* 68, 2016–2023.

27. van Alphen L B et al. (2008) Active migration into the subcellular space precedes *Campylobacter* *jejuni* invasion of epithelial cells. *Cell. Microbiol.* 10, 53–66.

28. Spohn G, Scarlato V (2001) *Motility, Chemotaxis, and Flagella*. *Helicobacter pylori: Physiology and Genetics*.

29. Nothaft H, Szymanski C M (2013) Bacterial protein n-glycosylation: New perspectives and applications. *J. Biol. Chem.* 288, 6912–6920.

30. Rubin E J, Trent M S (2013) Colonize, evade, flourish. *Gut Microbes* 4, 439–453.

31. Hanna A, Berg M, Stout V, Razatos A (2003) Role of capsular colanic acid in adhesion of uropathogenic *Escherichia* *coli*. *Appl. Environ. Microbiol.* 69, 4474–4481.

32. Hug I, Feldman M F (2011) Analogies and homologies in lipopolysaccharide and glycoprotein biosynthesis in bacteria. *Glycobiology* 21, 138–151.

33. Monteville M R, Yoon J E, Konkel M E (2003) Maximal adherence and invasion of INT 407 cells by *Campylobacter* *jejuni* requires the CadF outermembrane protein and microfilament reorganization. *Microbiology* 149, 153–165.

34. Onozato J et al. (2009) Cloning, sequencing and expression of full-length *Campylobacter* invasion antigen B gene operon from *Campylobacter* *lari*. *J. Basic Microbiol.* 49, 342–349.

35. Assié A et al. (2016) A specific and widespread association between deep-sea *Bathymodiolus* mussels and a novel family of Epsilonproteobacteria. *Environ. Microbiol. Rep.* 8, 805–813.

36. Bankevich A, Nurk S, Antipov D, Gurevich A a., Dvorkin M, Kulikov AS, et al. SPAdes: A New Genome Assembly Algorithm and Its Applications to Single-Cell Sequencing. *J Comput Biol* 2012; **19**: 455–477.
